# Iron-loaded deferiprone can support full hemoglobinization of cultured red blood cells

**DOI:** 10.1038/s41598-023-32706-1

**Published:** 2023-04-28

**Authors:** Joan Sebastián Gallego-Murillo, Nurcan Yağcı, Eduardo Machado Pinho, Sebastian Aljoscha Wahl, Emile van den Akker, Marieke von Lindern

**Affiliations:** 1 Department of Hematopoiesis, Sanquin Research and Landsteiner Laboratory, Amsterdam University Medical Center (UMC), Amsterdam, The Netherlands; 2 Department of Biotechnology, Faculty of Applied Sciences, Delft University of Technology, Delft, The Netherlands; 3 Department of Bioengineering, Faculty of Engineering, University of Porto, Porto, Portugal; 4 Present Address: Meatable, Alexander Fleminglaan 1, 2613AX Delft, The Netherlands; 5 Present Address: Lehrstuhl Für Bioverfahrenstechnik, Friedrich-Alexander Universität Erlangen-Nürnberg, Paul-Gordan-Str. 3, 91052 Erlangen, Germany

**Keywords:** Biotechnology, Haematopoietic stem cells

## Abstract

Iron, supplemented as iron-loaded transferrin (holotransferrin), is an essential nutrient in mammalian cell cultures, particularly for erythroid cultures. The high cost of human transferrin represents a challenge for large scale production of red blood cells (RBCs) and for cell therapies in general. We evaluated the use of deferiprone, a cell membrane-permeable drug for iron chelation therapy, as an iron carrier for erythroid cultures. Iron-loaded deferiprone (Def_3_·Fe^3+^, at 52 µmol/L) could eliminate the need for holotransferrin supplementation during in vitro expansion and differentiation of erythroblast cultures to produce large numbers of enucleated RBC. Only the first stage, when hematopoietic stem cells committed to erythroblasts, required holotransferrin supplementation. RBCs cultured in presence of Def_3_·Fe^3+^ or holotransferrin (1000 µg/mL) were similar with respect to differentiation kinetics, expression of cell-surface markers CD235a and CD49d, hemoglobin content, and oxygen association/dissociation. Replacement of holotransferrin supplementation by Def_3_·Fe^3+^ was also successful in cultures of myeloid cell lines (MOLM13, NB4, EOL1, K562, HL60, ML2). Thus, iron-loaded deferiprone can partially replace holotransferrin as a supplement in chemically defined cell culture medium. This holds promise for a significant decrease in medium cost and improved economic perspectives of the large scale production of red blood cells for transfusion purposes.

## Introduction

Iron is an essential nutrient for all eukaryotic microorganisms. Erythroid precursors, however, have exceptionally high iron requirements to facilitate hemoglobin synthesis during the generation of red blood cells (RBCs). A single RBC contains approximately 300 million molecules of hemoglobin (≈30 pg), associated with 1.2 × 10^9^ Fe^3+^ ions^1^. In healthy humans, 65–75% of all body iron is present as hemoglobin in RBCs^2^. Free ferrous iron Fe^2+^ can lead to the production of toxic radicals via the Fenton reaction^3^. Therefore, plasma iron is transported bound to transferrin (Tf). Human transferrin is a single chain glycoprotein with a molecular weight of ~ 80 kDa. It has two distinct iron-binding lobes (N- and C-lobe). The binding of Fe^3+^ to Tf is sequential, resulting in four possible Tf species: iron-free Tf (aTf: apotransferrin), diferric Tf (hTf: holotransferrin), and two monoferric Tf forms depending on which lobe contains the Fe^3+^ ion (mTf_N_ and mTf_C_ for the species with the iron ion in the N- or C-lobe, respectively)^4^.

High expression of the transferrin receptor 1 (TFR1; CD71) on erythroblasts facilitates their increased uptake of hTf for heme synthesis. Iron deficiency reduces the yield of burst-forming erythroid-units in early stages of ex vivo erythropoiesis^5^, and of reticulocytes during terminal erythroid differentiation^6^. Assuming that hTf is the sole source of iron, ~ 80 pg of hTf needs to be internalized for a single fully-hemoglobinized RBC, corresponding to ~ 160 g hTf for a single transfusion unit of RBCs (2 × 10^12^ RBCs). Transferrin is endocytosed upon binding to TFR1. Fe^3+^ ions are released at the low endocytic pH, reduced to Fe^2+^ by STEAP3, transported to mitochondria by direct endosome-mitochondria association, and ultimately incorporated into heme^7^. Excess iron is bound to cytosolic ferritin^8^. Vesicles containing aTf still bound to TFR1 recycle to the cell surface where aTf is released. In the body, the released aTf can bind new Fe^3+^ ions via direct transfer from ceruloplasmin ferroxidase^9^. In addition to TFR1, erythroblasts also express TFR2 which associates with the erythropoietin receptor (EpoR)^10,11^. Transferrin-dependent internalization of TFR2 modulates the Epo response, particularly at suboptimal Epo levels^12,13^.

The expression level of key regulatory proteins involved in iron uptake, storage and export is controlled by the RNA-binding proteins Iron regulatory protein-1 and -2 (IRP1/2)^14^. IRP1 binding to Fe–S clusters changes its conformation, and inhibits binding to iron-responsive elements (IREs) in the untranslated regions of mRNAs. For iron uptake proteins such as TFR1 and DMT1, IRP1 binding to IREs in the 3’ UTR of respective mRNAs increases mRNA stability^15^. By contrast, IRP1 binding to the IREs in the 5’ UTR of mRNAs encoding proteins involved in iron storage and export (e.g. ferritin and ferroportin) decreases their translation^16,17^. Iron deficiency decreases heme levels, which activates Heme-regulated eIF2α kinase (HRI), resulting in phosphorylation of eIF2 and a global inhibition of protein translation^18–20^. At the level of erythropoiesis, lack of iron causes microcytic anemia in vivo^21^, and decreases the proliferation and differentiation of erythroblast cultures ex vivo ^6^.

In serum-free cell culture systems, high levels of hTf (1000 µg/mL) are added during terminal differentiation^22,23^. Currently, most of the Tf used in cultures is purified from human plasma. However, there is a growing need to transition towards animal-free media components to ensure safety of the final product. Production of recombinant Tf in *Escherichia coli*, insects, yeast, rice and BHK cells represents an alternative, but is still not cost-effective for the ex vivo production of RBCs^24^.

Several small molecules have been reported to chelate and deliver iron into the cells. Iron-loaded PIH (pyridoxal isonicotinoyl hydrazone) restored proliferation and differentiation in several cell lines in absence of hTf by directly crossing the cellular membrane^25–27^. Supplementation of iron-loaded hinokitiol was also shown to restore proliferation and hemoglobinization in DMT1-deficient cells in transferrin-free medium, although its effectiveness seems to depend on large iron gradients across the cellular membrane^28^. More recently, a low Tf culture medium for cRBC production has been reported, in which supplementation with Fe^3+^-EDTA supports reloading of Tf, which can then be re-utilized by the cells^29^.

Chelators used to treat iron overload in vivo have a high affinity for iron that is lower than transferrin, but allows to scavenge all non-transferrin-bound iron (NTBI) to prevent oxidative damage^30,31^. Among them is deferiprone (1,2-dimethyl-3-hydroxypyridin-4-one), a bidentate alpha-keto hydroxypyridine molecule that binds iron with a stoichiometry of 3:1 (Def_3_·Fe^3+^) at physiological conditions^32^. Its ability to cross the cell membrane and mobilize intracellular iron is the principle of combined chelation therapy, in which deferiprone scavenges intracellular iron and transfers it to a second non-permeable chelator such as deferoxamine^33,34^.

In this study, we evaluated the potential of iron-loaded deferiprone (Def_3_·Fe^3+^) to replace the supplementation with high levels of hTf in cultures of erythroid precursors for the ex vivo production of RBCs. We demonstrated that Def_3_·Fe^3+^ can recharge aTf to hTf. Medium supplementation with Def_3_·Fe^3+^ alone was sufficient to sustain efficient ex vivo production of fully hemoglobinized RBCs with an oxygen binding capacity comparable to peripheral blood RBCs. Excess Def_3_·Fe^3+^ showed no sign of toxicity in erythroblast proliferation and differentiation. Additionally, the use of Def_3_·Fe^3+^ as the only iron source in serum-free medium was sufficient to sustain the proliferation of other transferrin-dependent mammalian cell lines.

## Results

### Iron-loaded deferiprone supports erythroid differentiation at low holotransferrin concentrations

**Figure 1:**
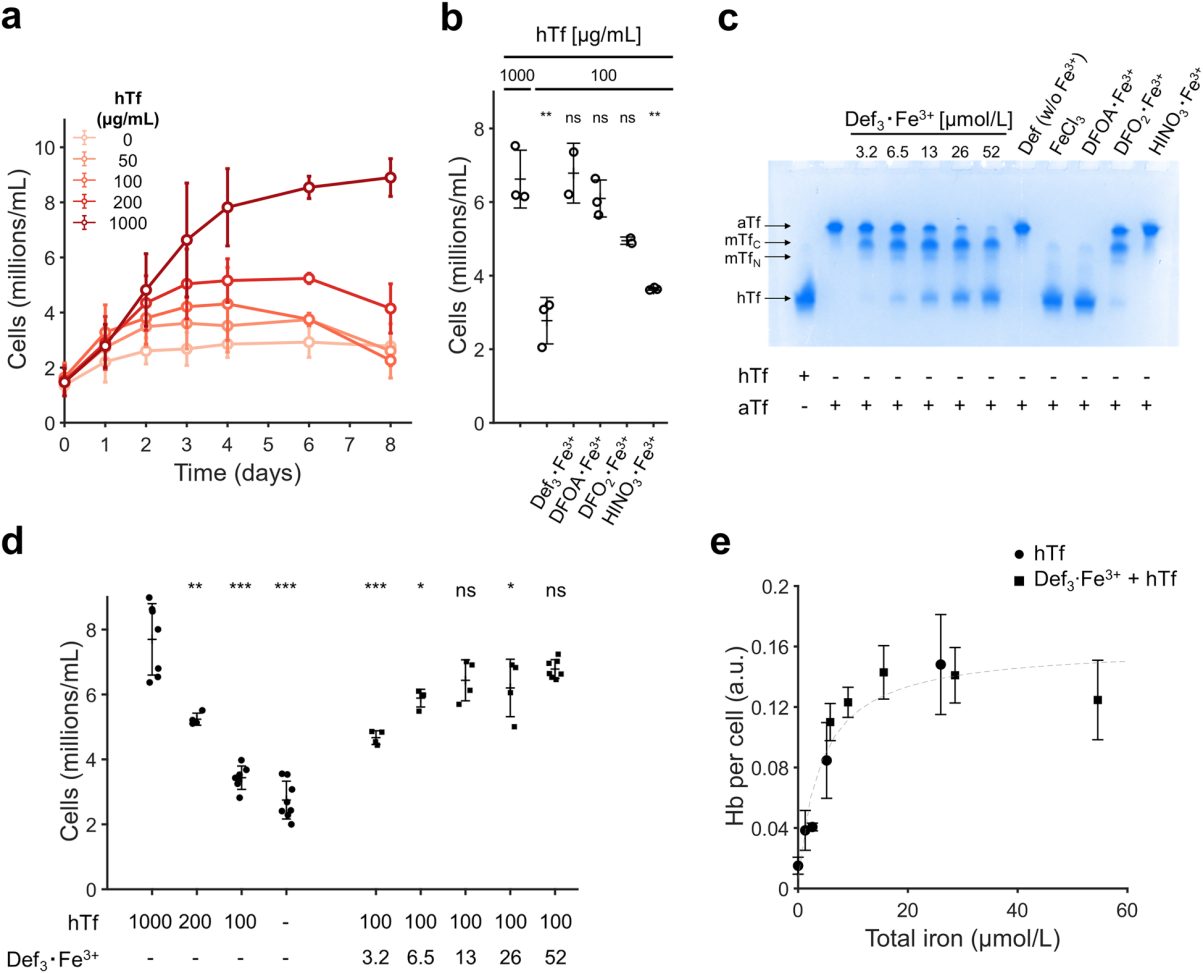
Deferiprone supplementation restores efficient differentiation of erythroblasts in transferrin-limited medium. Erythroblasts were expanded from PBMCs for 10–12 days, and subsequently seeded in differentiation medium at a starting cell concentration of 1.5–2.0 million cells/mL. (**a**) Cells were seeded with decreasing holotransferrin concentrations (1000, 200, 100, 50 and 0 µg/mL). Cell number was determined at indicated days. (**b**) Cell concentration at day 7 of differentiation in medium with low hTf (100 µg/mL), with or without 52 µmol/L Fe^3+^ associated with one of four chelators (deferiprone = Def; deferoxamine = DFOA; deferasirox = DFO; hinokitiol = HINO). (**c**) 1000 µg/mL apotransferrin was incubated for 16 h with iron-loaded chelators (52 µmol/L of chelated Fe^3+^, unless indicated). All samples were incubated overnight in PBS at 37 °C (pH = 7.4). All samples were mixed 1:1 with Novex™ TBE-Urea gel sample buffer, loaded in a Novex™ 6% TBE-urea gel (3.0 µg of Tf per well; Invitrogen; Waltham, MA), and run for 2.25 h at 150 V. Gels were stained with Coomassie. Full uncropped gel image is displayed. Purified holo-transferrin (first lane) and apo-transferrin (second lane) function as size markers. (**d**) Cell concentration after 6 days of differentiation in medium with hTf (values in µg/mL) and Def_3_·Fe^3+^ (values in µmol/L) as indicated. (**e**) Hemoglobin content per cell after 4 days of differentiation at increasing iron concentration in cells cultured using hTf as sole iron source (filled circle), or Def_3_·Fe^3+^ plus 100 µg/mL hTf (filled square). A hyperbolic dose–response curve is fitted on the data (Hb = Hb_max_ × [Fe^3+^] × (EC_50_ + [Fe^3+^])^−1^). All data is displayed as mean ± SD (error bars; n ≥ 3). Significance is shown for the comparison with 1000 µg/mL hTf (unpaired two-tailed two-sample Student’s *t*-test).

As reference for the different iron supplementation strategies, we first tested different holotransferrin concentrations during erythroblast differentiation, the culture stage in which cells have the highest iron requirements. Twelve days post-seeding PBMCs in expansion medium, erythroblasts were cultured in differentiation medium supplemented with decreasing concentrations of hTf (1000, 200, 100, 50 and 0 µg/mL; equivalent to 26, 5.2, 1.3 and 0 µmol/L of chelated Fe^3+^, respectively). At 1000 µg/mL hTf, erythroblasts proliferated for 3–4 days followed by cell growth arrest at the final stage of differentiation^22^. A gradual decrease of hTf concentrations resulted in a gradual decrease in cell yields, in accordance with previously described data (Fig. 1a)^6^. Erythroblast differentiation is accompanied by a progressive loss of cell volume to reach the cell size of erythrocytes. Suboptimal hTf concentrations led to a faster decrease in cell size during the first 4 days compared with 1000 µg/mL hTf, and a consistently lower cell volume that was still present after 8 days of culture (Fig. S1a). This seems to be in accordance with microcytic anemia associated with iron deficiency. However, cultured reticulocytes are generally larger compared to primary erythrocytes, and also reticulocytes cultured at low iron concentrations remain larger compared to primary erythrocytes. Thus, it is not clear how low or high concentrations of iron result in aberrantly sized cultured reticulocytes. Hemoglobin (Hb) levels per cell were low at the start of differentiation, and rapidly increased dependent on the hTf concentration (Fig. S1b).

hTf is internalized upon binding to TFR1. After releasing Fe^3+^, iron-depleted transferrin (apotransferrin; aTf) is recycled back to the cell membrane together with the TFR1. We tested whether supplementation of culture medium with an iron-loaded chelator can sustain differentiation of erythroblasts at low hTf concentrations. Four chelators loaded with iron (52 µmol/L chelated Fe^3+^) were added to medium with a growth-limiting hTf concentration (100 µg/mL). Supplementation with iron-loaded deferiprone (Def_3_·Fe^3+^) or deferoxamine (DFOA·Fe^3+^) enabled proliferation similar to 1000 µg/mL of hTf. To a lesser extent also deferasirox (DFO_2_·Fe^3+^) and hinokitiol (HINO_3_·Fe^3+^) rescued growth recovery (Fig. 1b).

To test the ability of these chelators to reload aTf, 1000 µg/mL aTf was incubated with 52 µmol/L of chelated iron at 37 °C overnight and subjected to urea-PAGE gel electrophoresis. The mobility of aTf is lower compared to hTf, while the monoferric transferrin forms (mTf_C_, mTf_N_) have an intermediate migration speed. Deferasirox and hinokitiol showed a low Tf saturation level, while both deferiprone and deferoxamine could reload most aTf (Fig. 1c). Soluble iron, added as FeCl_3_, was also able to reload the majority of aTf, with minor amounts of both mTf forms present. Although deferiprone resulted in more mTf compared to deferoxamine, the former was favored for stability^35^ and cost-effectiveness.

Decreasing deferiprone concentrations reduced the final saturation level of Tf (Fig. 1c). To further test the effect of iron-loaded deferiprone supplementation (Def_3_·Fe^3+^) on iron-limited erythroid differentiation, erythroblasts were seeded in differentiation medium supplemented with 100 µg/mL hTf and Def_3_·Fe^3+^ at concentrations between 3.2 and 52 µmol/L (equivalent to 125 and 2000 µg/mL hTf, respectively). Def_3_·Fe^3+^ improved cell growth dependent on the Def_3_·Fe^3+^ concentration (Fig. 1d).

In differentiation cultures, most of the hemoglobin is synthesized in the first 2–4 days of culture, and hemoglobin content per cell is strongly dependent on the hTf concentration in the medium (Fig. S1b). At day 4, the hemoglobin content of cultured cells showed saturation kinetics (hyperbolic) as a function of the media iron content. Of note, cells cultured in absence of hTf had a tenfold decrease in hemoglobin (Hb) mass per cell compared to the highest hTf dose tested (Fig. 1e). This dose–response behavior was also observed when using 100 µg/mL hTf with increasing deferiprone concentrations, with a maximal response using > 13 µmol/L Def_3_·Fe^3+^. The same iron-dependent response was seen for hemoglobin accumulation (Hb intracellular concentration; Fig. S1c-d), with no difference observed between Def_3_·Fe^3+^ concentrations > 13 µmol/L and medium containing 1000 µg/mL hTf.

The data suggests that Def_3_·Fe^3+^ supplementation can reload apotransferrin generated during culture. To validate this assumption, we calculated the concentrations of transferrin and deferiprone species assuming equilibria at culture conditions (pH = 7.4, 3.5 mmol/L HCO_3_^−^; Fig. S2). For transferrin, the association equilibrium constants of 7.0 × 10^22^ L/mol and 3.6 × 10^21^ L/mol for the binding of the first and second Fe^3+^ ion were assumed, respectively^36^. For deferiprone, the global stability constants (log β) for the Def‧Fe^3+^, Def_2_‧Fe^3+^ and Def_3_·Fe^3+^ complexes were assumed to be 15.01, 27.30 and 37.43, respectively^32^. It was assumed that iron association and dissociation kinetics with Tf and Def dominate iron concentration in the extracellular space, and that this is faster than the net utilization rate of iron in the intracellular space. As a model condition, a constant net average iron uptake rate (inflow + iron export from the cells) of 1.7 × 10^–7^ mol Fe^3+^/L‧h was assumed, corresponding to the production of 10 × 10^6^ hemoglobinized cells per mL of culture (1 cell = 300 million Hb molecules) in the first 4 days of culture, as observed in Fig. S1b. The calculated time profiles for the concentrations of the different transferrin and deferiprone species indicated that the addition of 26 or 52 μmol/L Def_3_‧Fe^3+^ to 100 mg/L hTf at the start of differentiation results in higher holotransferrin availability (percentage of total Tf) and lower apotransferrin levels, and thereby to a potentially better iron delivery compared to the addition of 1000 mg/L hTf alone (Fig. S2c). Although this simplified model does not consider direct transfer of Fe^3+^ from Def_3_‧Fe^3+^ to transferrin, the chemical equilibria simulations support the observation that Def_3_·Fe^3+^ can reload iron-depleted transferrin, even by assuming the leaching of Fe^3+^ as the sole mechanism of iron transfer between the two chelators.

### Iron-loaded deferiprone can replace holotransferrin supplementation in differentiation

**Figure 2:**
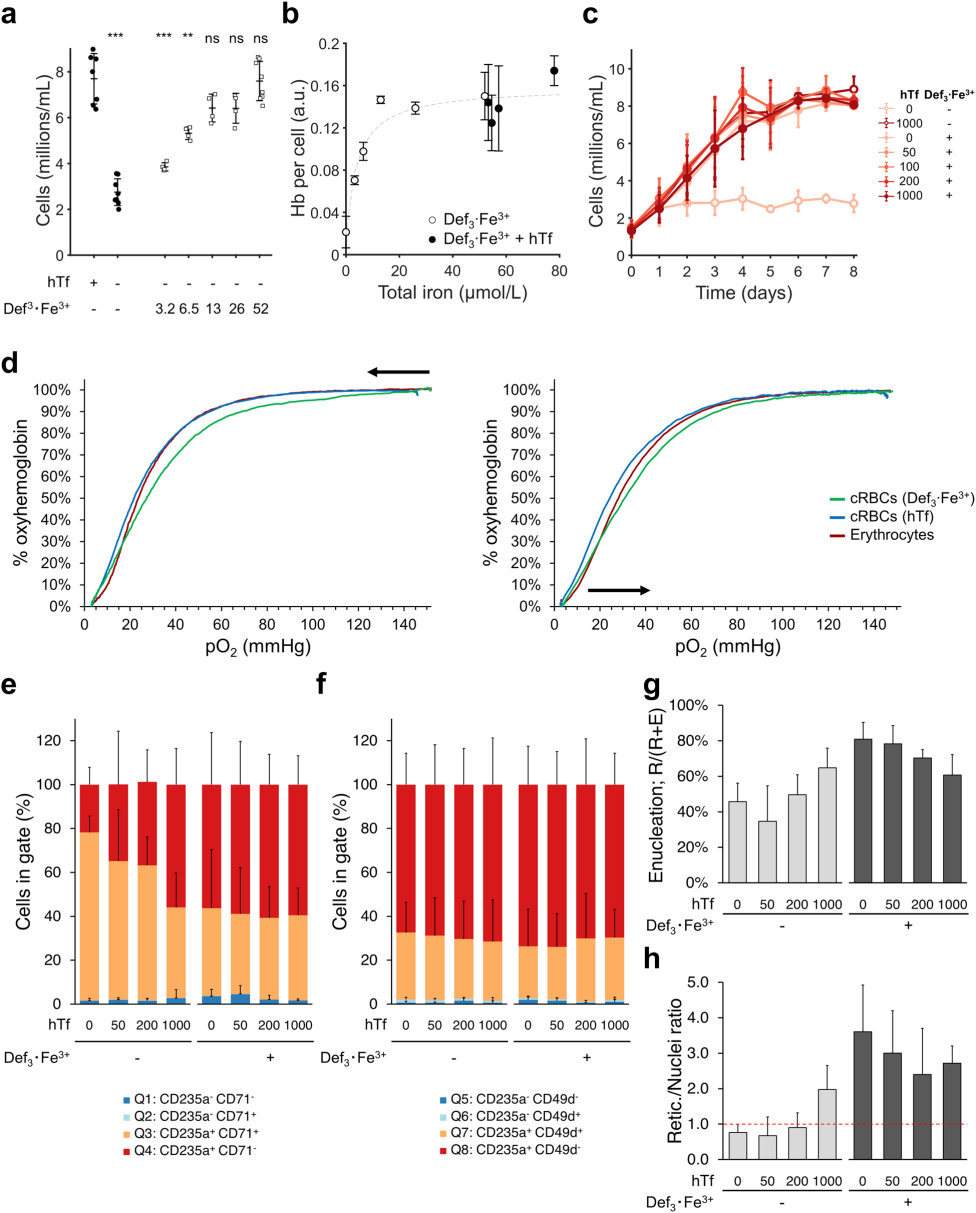
Deferiprone can replace holotransferrin supplementation in erythroblast differentiation cultures. Erythroblast were expanded from PBMCs for 10–12 days, and subsequently seeded in differentiation medium. (**a**) Cell concentration after 6 days of differentiation in medium without hTf supplementation, with varying levels of Def_3_·Fe^3+^ (concentrations indicated in µmol/L). (**b**) Hemoglobin content of cells after 4 days in differentiation in medium using Def_3_·Fe^3+^ as sole iron source (empty circle), or with varying hTf concentrations (1000, 500, 200, 100, 50 µg/mL) in the presence of 52 µmol/L Def_3_·Fe^3+^ (filled circle). (**c**) Cell concentration of erythroblasts seeded in medium with or without 52 µmol/L Def_3_·Fe^3+^ (filled and empty symbols, respectively), plus a decreasing concentration of hTf (values in µg/mL). (**d**) Oxygen dissociation (left) and association (right) curves measured by a HEMOX analyzer for peripheral blood erythrocytes (red), and reticulocytes cultured using either 1000 µg/mL hTf (blue) or 52 µmol/L Def_3_·Fe^3+^ (green) as iron supplement. (**e**–**h**) Cells, differentiated for 10 days in presence of hTf and/or Def_3_·Fe^3+^ as indicated, were stained with CD235 plus CD71 (**e**), CD235a plus CD49d (**f**), or DRAQ5 (cell permeable DNA stain; **g,** **h**). Relative cell numbers per quadrant were calculated (gating strategy available in Fig. S3a). (**g**) Enucleation percentage of erythroid cells, and (**h**) ratio of reticulocytes versus pyrenocytes (extruded nuclei) were calculated from the forward scatter and DRAQ5 staining. A retic./nuclei ratio > 1 means more reticulocytes than nuclei. Conditions labeled as 0 µg/mL hTf correspond to differentiation medium without hTf supplementation. Trace levels of hTf are present (< 0.2 µg/mL) due to the plasma used in the differentiation medium (5%). All data is displayed as mean ± SD (error bars; n ≥ 3). Significance is shown for the comparison with the 1000 µg/mL hTf condition (unpaired two-tailed two-sample Student’s *t*-test).

Due to its lipophilic nature and small size, deferiprone is expected to be cell permeable and able to redistribute iron between cell types independent of ferroportin and transferrin^37,38^. We tested whether the larger Def_3_·Fe^3+^ complex can support erythroid differentiation in absence of transferrin supplementation (Tf concentration < 0.2 µg/mL due to the 5% plasma used in differentiation medium^39^). Expanded erythroblast cultures (day 12) were reseeded at 1.5 × 10^6^ cells/mL in differentiation medium with increasing concentrations of Def_3_·Fe^3+^ as sole iron source. At day 6 of differentiation, 3 times higher cell numbers were obtained using > 13 µmol/L Def_3_·Fe^3+^ compared with no hTf supplementation and no Def_3_·Fe^3+^ cultures (Fig. 2a). Similar dose–response curves were obtained for total hemoglobin per cell (Fig. 2b). The addition of increasing hTf concentrations to optimal concentrations of Def_3_·Fe^3+^ (52 µmol/L) did not further increase cell number or hemoglobin content (Fig. 2b–c). Interestingly, the combination of high levels Def_3_·Fe^3+^ together with a high concentration hTf did not lower cell numbers, suggesting that Def_3_·Fe^3+^ is not toxic to erythroblasts.

Oxygen association and dissociation are crucial for RBC function. Reticulocytes obtained from differentiation cultures supplemented with 52 µmol/L Def_3_·Fe^3+^ had similar hemoglobin oxygen dissociation and association curves compared to peripheral blood RBCs and reticulocytes obtained in medium supplemented with hTf, with a slightly higher P_50_ (blood = 26.8 mmHg, hTf = 24.2 mmHg, deferiprone = 28.0 mmHg; calculated from oxygenation profile; Fig. 2d).

Flow cytometry studies were performed to evaluate the effect of hTf and Def_3_·Fe^3+^ on the erythroblast maturation level. During ex vivo differentiation cultures, erythroblast acquire CD235 (Glycophorin A), and eventually lose CD71 (TFR1) expression^22^. Expression of CD71 may not be a reliable differentiation marker when evaluating the effect of hTf and Def_3_·Fe^3+^ supplementation as it is upregulated at low iron availability^40^. At day 10 of differentiation, cells had a high expression of CD235 under all tested conditions (gating strategy in Fig. S3a). In contrast, CD71 expression levels decreased with increasing iron availability, independent of whether iron is presented as hTf or Def_3_·Fe^3+^ (Fig. 2e; Fig. S3b). CD49d (integrin alpha 4) is an early differentiation marker^41^ expressed independent of iron metabolism regulation. Regardless of the hTf or Def_3_·Fe^3+^ concentration, 30–40% of all cells had a CD235^+^ CD49d^+^ phenotype, suggesting that deferiprone supplementation did not delay erythroid differentiation (Fig. 2f).

The availability of chelated iron has been reported to affect erythroblast enucleation^42^. Enucleation efficiency of the cultures was evaluated using the nuclear stain DRAQ5 and cell size by flow cytometry (Fig. S3a). The ratio between enucleated (R) and total cells (R + E) indicates the enucleation ratio. High hTf concentrations led to a higher enucleation efficiency (65%) compared with suboptimal hTf concentrations (0 µg/mL hTf: 46%; 50 µg/mL hTf: 35%; 200 µg/mL hTf: 50%). Addition of 52 µmol/L Def_3_·Fe^3+^ increased terminal enucleation to 80% in the absence of hTf (Fig. 2g). In addition, Def_3_·Fe^3+^ increased the ratio of reticulocytes over nuclei, indicating a positive effect on reticulocyte stability (Fig. 2h). Collectively, these results suggest that Def_3_·Fe^3+^ can replace hTf as medium supplement in differentiation cultures, maintaining high cell yields and enucleation efficiency, hemoglobin content and oxygen carrying capacity.

### Def_3_·Fe^3+^ supplementation prevents molecular responses to iron deficiency

**Figure 3:**
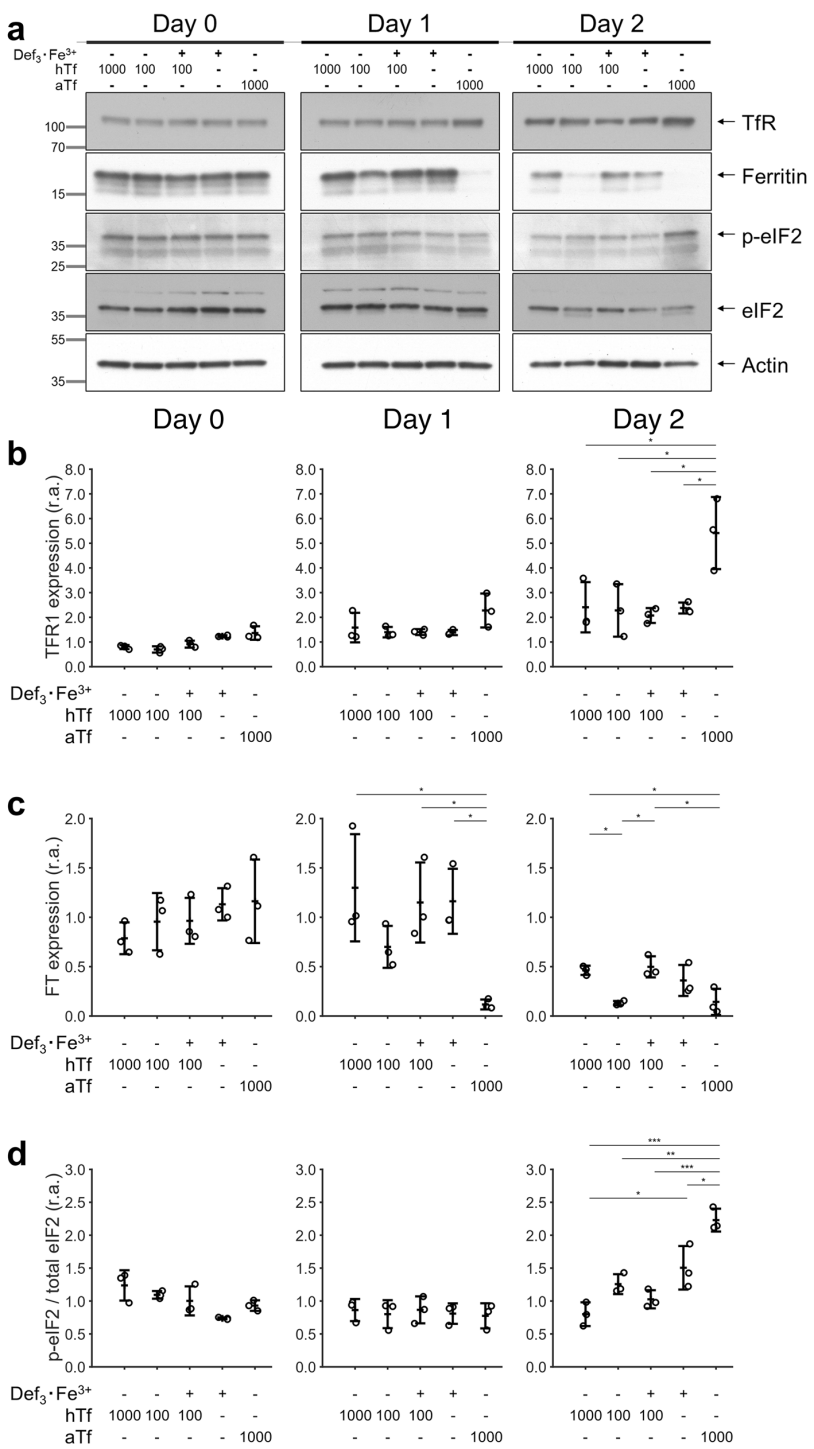
Recovery of iron regulation metabolism using deferiprone in differentiation. Erythroblasts were expanded from PBMCs for 10–12 days, and subsequently seeded in differentiation medium supplemented with hTf or aTf (values µg/mL) or Def_3_·Fe^3+^ (52 µmol/L). Cells were harvested at seeding (Day 0), and after 1 and 2 days of culture. (**a**) Western blots of one representative donor showing the expression level of proteins involved in iron metabolism regulation. Actin was used as housekeeping protein for relative quantification of protein abundance. Position of size markers is indicated in kDa. Membrane images were cropped by timepoint for clarity of the presentation, indicated by the dividing black lines and white space in between. Blots were cut in separate size intervals. The processing of blots and raw scans are shown in Fig. S4. (**b**–**c**) Relative abundance (r.a.) of transferrin receptor (TFR1) and ferritin. Relative protein levels were calculated using actin signal, followed by normalization using the average of each donor at the start of the culture (day 0). (**d**) Level of the phosphorylated form of eIF2 (p-eIF2) relative to the total levels of eIF2. In order to compare different experiments, the relative p-eIF2/eIF2 ratio was normalized using the average of each donor at the start of the culture (day 0). Conditions labeled as 0 µg/mL hTf correspond to differentiation medium without hTf supplementation. Data is displayed as mean ± SD (error bars; n = 3). Significance is shown for the comparison with 1000 µg/mL hTf (unpaired two-tailed two-sample Student’s *t*-test with Helm-Bonferrin correction for multiple comparisons).

Next, we studied whether iron supplementation by Def_3_·Fe^3+^ and hTf similarly controlled cellular iron metabolism in erythroblasts. The IRP1/2-dependent expression of ferritin and TFR1, and HRI-dependent phosphorylation of eIF2 was measured during the first 2 days of differentiation in presence of high and low levels of hTf or Def_3_·Fe^3+^ (Fig. 3a and Fig. S4). The expression of TFR1 was upregulated in presence of apotransterrin. This is in accordance with IRP stabilizing TFR1 mRNA in iron-limiting conditions (Fig. 2e). Low iron concentrations did not significantly affect TFR1 expression, possibly due to the stability and recycling of the TFR1 (quantification: Fig. 3b).

Ferritin synthesis, in contrast, is inhibited upon IRP binding to ferritin mRNA^17^. Ferritin levels remained high in presence of high total iron concentrations (1000 µg/mL hTf, or 52 µmol/L Def_3_·Fe^3+^), but were reduced at low or absent iron levels (100 µg/mL hTf, or apotransferrin) (Fig. 3c). Finally, high levels of p-eIF2 were observed in cultures with iron limitation (Fig. 3d; day 2). Def_3_·Fe^3+^ supplementation restored the low phosphorylation levels of eIF2 detected in cultures with high hTf concentration. Together, our results suggest that iron supplementation to erythroblast differentiation cultures either by Def_3_·Fe^3+^ or hTf is fully interchangeable at the molecular level.

### Medium containing iron-loaded deferiprone can sustain expansion of erythroblasts

**Figure 4:**
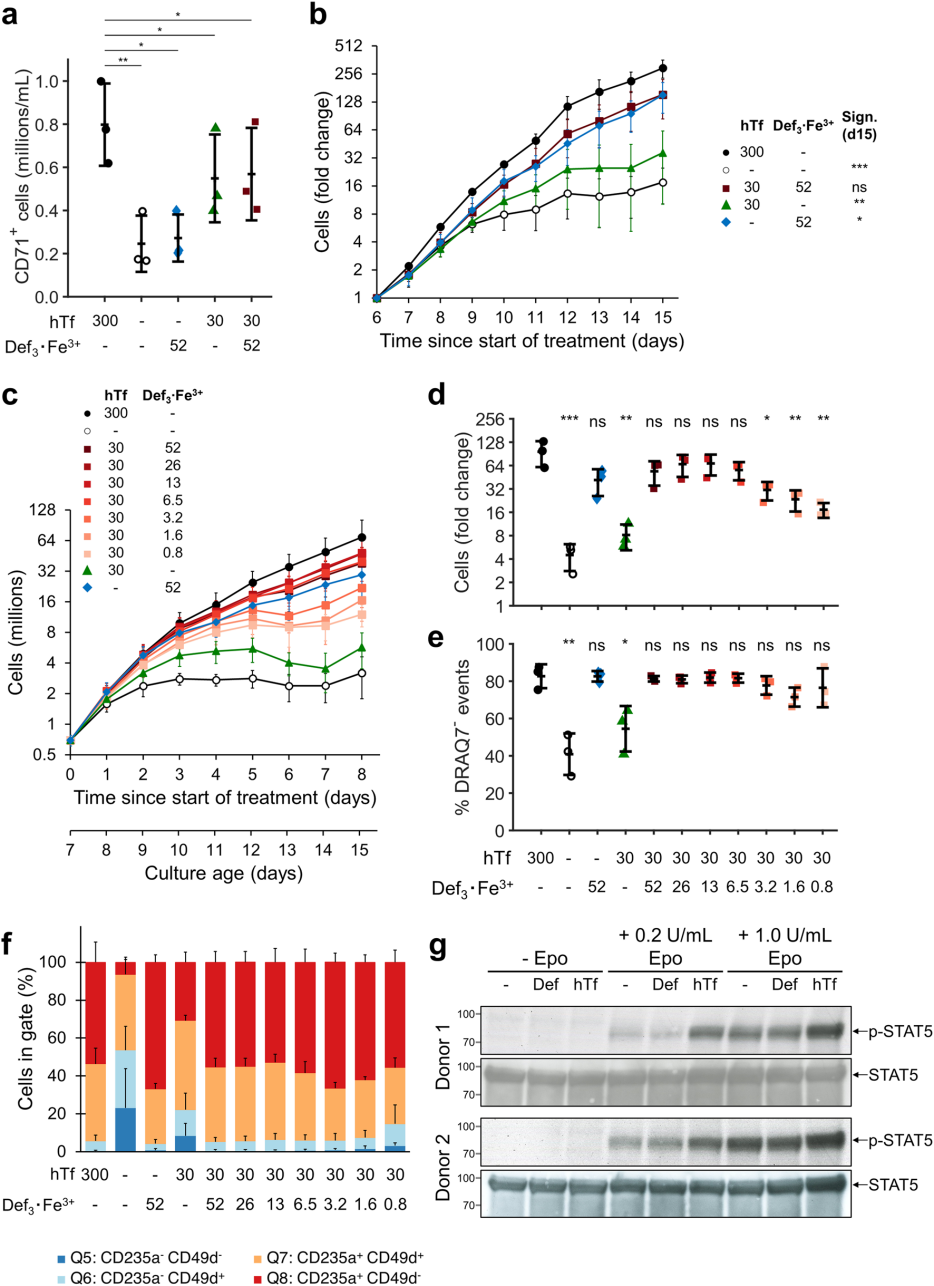
Deferiprone supplementations sustains erythroblast expansion. Adult PBMCs were isolated (day 0) and cultured in expansion medium at a starting cell concentration of 1 million cells/mL using hTf (values in µg/mL) in the presence or absence of Def_3_·Fe^3+^ (52 µmol/L). (**a**) Total number of CD71^+^ cells was determined on day 6 after PBMC isolation (combining cell count and CD71 + frequency by flow cytometry; data available in Fig. S4). (**b**) Erythroblast cell concentration was monitored for additional 9 days of culture, with daily medium refreshment. Fold change in cell number was calculated using the measured number of erythroblasts at day 6. (**c**–**f**) Erythroblasts were expanded from PBMCs in expansion medium with hTf as iron source (300 µg/mL) for 7 days, and subsequently reseeded in medium with varying hTf concentrations (300, 30, 0 µg/mL) in the presence of Def_3_·Fe^3+^ (0.8–52 µmol/L Def_3_·Fe^3+^). Cultures were maintained for 8 days (15 days since PBMC isolation) during which daily medium refreshments were performed (**c**). After 8 days of culture, cell number fold change was calculated relative to the start of treatment (**d**). Cells were stained with DRAQ7 (cell impermeable DNA stain; **e**), and CD235a plus CD49d (**f**). Relative cell numbers per quadrant were calculated (representative density plots available in Fig. S5). (**g**) Analysis of the effect of Def_3_·Fe^3+^ on Epo signaling. Erythroblasts were cultured with hTf (300 µg/mL), Def_3_·Fe^3+^ (52 µM) or no iron, withdrawn from Epo for 3 h, and restimulated (0.2 or 1.0 U/mL EPO). Lysates were subjected to western blot analysis using phospho-STAT5 and total-STAT5 antibodies. All 9 samples (3 Epo concentrations × 3 iron supplementation treatments) were run in the same gel. Original uncropped membrane images for both donors are available in Fig. S7. Data in panels (**a**–**f**) is displayed as mean ± SD (error bars; n = 3). Significance is shown for the comparison with 300 µg/mL hTf (unpaired two-tailed two-sample Student’s *t*-test). Data for panels b-e available in Supplementary Table S1.

Iron requirements are especially high during erythroblast differentiation when hemoglobin is synthesized. However, cells do require iron as constituent of essential metalloproteins, such as cytochromes and some peroxidases^43^. We tested if deferiprone could also replace hTf supplementation during the first phase of our culture protocol, when hematopoietic stem and progenitor cells commit to the erythroid lineage, and subsequently when committed erythroblasts proliferate but hemoglobin production remain low. Erythroid cultures were established from peripheral blood mononuclear cells (PBMCs) in serum-free medium supplemented with the standard hTf concentration for culturing of non-hemoglobinized cells (300 µg/mL hTf), or with a 10× lower hTf concentration, in presence or absence of Def_3_·Fe^3+^ (52 µmol/L). The presence of hTf (300 µg/mL) was required to obtain a culture of CD71^+^ cells from PBMCs (Fig. 4a; Fig. S3a-b), while deferiprone alone was not sufficient to establish an erythroid culture in the absence of hTf supplementation. At a lower hTf concentration (30 µg/mL) cultures yielded less CD71^+^ erythroblasts, which was unaffected if deferiprone was supplemented. As shown previously, CD71^+^ cells cultured in absence of hTf expressed CD71 at increased levels. In these early stages, deferiprone did not reduce CD71 levels to those observed with hTf (Fig. S5). Upon prolonged expansion of committed erythroblasts Def_3_·Fe^3+^, alone or supplemented to low hTf levels, resulted in the same erythroblast growth rate as when 300 µg/mL hTf was used (Fig. 4b).

To determine the optimal Def_3_·Fe^3+^ concentration to sustain proerythroblast proliferation once erythroid cultures are established, PBMCs were first expanded for 6 days in medium with hTf, followed by culture in medium with a suboptimal hTf level supplemented with Def_3_·Fe^3+^ at concentrations between 0.8 and 52 µmol/L (Fig. 4c). Maximum recovery required at least 6.5 µmol/L Def_3_·Fe^3+^ (Fig. 4d). Lack of iron, or low levels of iron (30 µg/mL hTf) increased the number of non-viable cells as detected by DRAQ7. Supplementation with Def_3_·Fe^3+^ recovered viability of the expansion cultures (Fig. 4e).

High CD71 (TfR) expression characterizes erythroblasts. During the expansion phase of erythroblast cultures, the CD71^high^ erythroblast population slowly gains CD235 expression from day 10 onwards ^22^. A CD71^mid^ CD235^−^ subpopulation, that was observed under iron-limited conditions (0 and 30 µg/mL hTf) and less prominently under low Def_3_·Fe^3+^ concentrations (1.6 and 0.8 µmol/L), indicates a non-erythroid cell population that remains due to the failure of erythroblast outgrowth (Fig. S6). The presence of these seemingly non-erythroid cells was also observed when following the expression of CD49d (Fig. 4f). High levels of Def_3_·Fe^3+^ enhanced the gain of CD235 and loss of CD49d to levels observed with 300 µg/mL hTf.

Because TFR2 associates with the EpoR, reduced internalization and recycling of TFR2/EpoR complex to the membrane was shown to modulate EpoR signaling^12,13^. To investigate whether Def_3_·Fe^3+^ supplementation interferes with EpoR signaling, erythroblasts were cultured in presence of hTf or Def_3_·Fe^3+^, deprived of Epo and restimulated (0.2 or 1.0 U/mL), all in presence of hTf or Def_3_·Fe^3+^. In accordance with previous reports, Epo-induced STAT5 phosphorylation was reduced in absence of hTf, and Def_3_·Fe^3+^ did not restore STAT5 phosphorylation (Fig. 4g and Fig. S7).

### Iron-loaded deferiprone can replace holotransferrin for other myeloid cell lines

**Figure 5:**
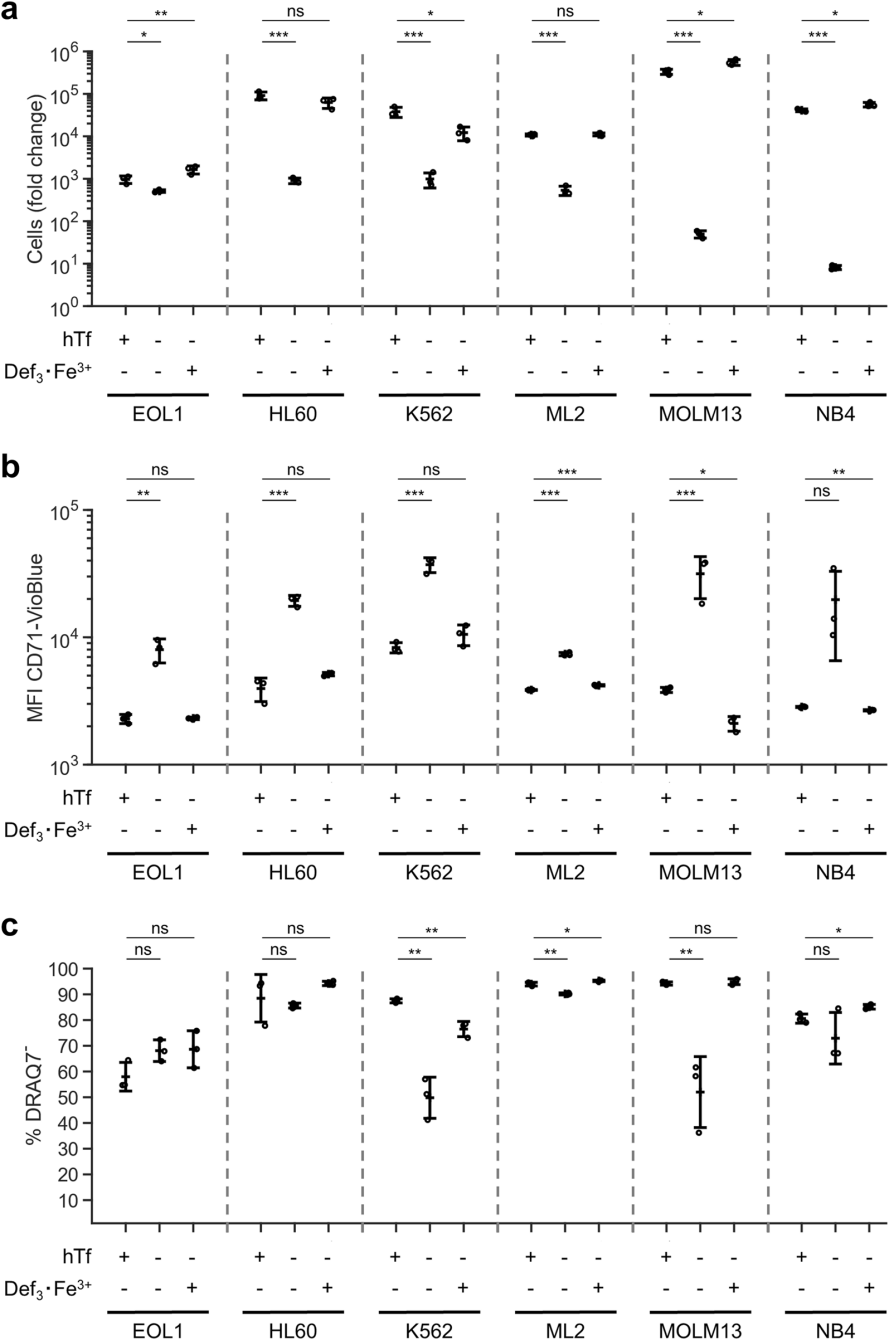
Deferiprone sustains expansion of selected myeloid cell lines. Myeloid cell lines were cultured in Cellquin medium supplemented or not with 300 µg/mL hTf or 52 µmol/L Def_3_·Fe^3+^ for 16 days. (**a**) Total cell number fold change relative to day 0. (**b**) Expression level of the transferrin receptor (CD71) determined by flow cytometry. MFI for corresponding isotypes is between 1 × 10^2^–3 × 10^2^. (**c**) Cells were stained with DRAQ7 (cell impermeable DNA stain) to quantify the viability of the cultures. Data is displayed as mean ± SD (error bars; individual datapoints are indicated; n = 3). Significance is shown for the comparison with 300 µg/mL hTf (unpaired two-tailed two-sample Student’s *t*-test).

As a limited number of cell lines are able to produce Tf, it is commonly used as supplement in chemically defined media (i.e. serum-free) for most mammalian cell cultures^44^. Thus, we evaluated the potential of Def_3_·Fe^3+^ to fully replace holotransferrin in cultures of the myeloid cell lines MOLM13, NB4, EOL1, K562, HL-60, and ML-2. Cells were seeded in media containing no iron, 300 µg/mL hTf or 52 µmol/L Def_3_·Fe^3+^. All cell lines could be expanded in our serum-free medium supplemented with hTf (Fig. 5a; Fig. S8). Lack of iron decreased the growth rate, with MOLM13 and NB4 cells showing a decrease of 4 orders of magnitude in cell numbers after 16 days of culture. EOL1 cultures seemed less sensitive to lack of hTf, but proliferation in presence of hTf was also low. Def_3_·Fe^3+^ recovered growth to levels comparable with only-hTf conditions. As observed in ex vivo erythroblast expansion and differentiation cultures, lack of hTf led to an upregulation of CD71 expression in all cell lines (Fig. 5b). Def_3_·Fe^3+^ supplementation restored CD71 expression levels and viability (Fig. 5c) comparable to that in hTf-only cultures.

## Discussion

Hemoglobin production requires a high uptake of iron in differentiating erythroblasts, which is supplied as hTf. We evaluated whether iron-loaded chelators can be a low-cost alternative for the high hTf supplementation levels required in ex vivo erythroid cultures. Our data show that deferiprone, a bidentate α-ketohydroxypyridine iron-specific chelator, could replace holotransferrin supplementation to produce large numbers of enucleated hemoglobinized cultured RBCs at concentrations as low as 26 μmol/L (Def_3_·Fe^3+^). Hemoglobin content and oxyhemoglobin dissociation dynamics of cultured RBCs (cRBCs) derived with Def_3_·Fe^3+^ or with hTf as sole iron source were similar.

We show that hemoglobin is expressed at similar levels, and is equally functional in reticulocytes cultured in presence of hTf or Def_3_·Fe^3+^. Iron-loaded chelators can serve as an iron pool to low amounts of transferrin still present in our cultures dependent on their affinity for iron. Chelators with a relatively low affinity for iron such as deferiprone (pFe^3+^: 19.3) will transfer Fe^3+^ to aTf (pFe^3+^: 22.3; pFe^3+^ value is defined as -log_10_ [Fe^3+^] with 1 μM total iron and 10 μM total ligand at pH 7.4). Instead, chelators with a pFe^3+^ larger than aTf such as deferoxamine mesylate (DFOA) and deferasirox (DFO; pFe^3+^  > 26) will deplete hTf from iron. By consequence, deferiprone is very well suited to stabilize Fe^3+^ in cell culture media and to transfer Fe^3+^ to aTF to enable uptake via TfR1 and TfR2.

Tf may be present due to, for instance, the use of plasma in differentiation cultures, or due to intracellular pools of transferrin from the preculture stage. Deferiprone, however, is able to permeate the cell and access labile iron pools in subcellular compartments possibly due to its low molecular weight (139.1 g/mol) and mild lipophilic nature (D_7.4_ = 0.17), as previously reported^45,46^. Deferiprone's ability to enter cells is not clearly required to enable iron transfer to Tf. However, we speculate that this known characteristic of deferiprone may enable a more robust redistribution of iron between cells in culture. Deferiprone can mobilize intracellular iron and render it available for hemoglobin synthesis^34^. Other iron-loaded chelators such as DFOA·Fe^3+^ can also function as substrate for heme synthesis instead of holotransferrin^47^. Excessive iron availability, however, may also cause oxidative damage^48,49^. High levels of Def_3_·Fe^3+^ (26–52 µmol/L) did not have a detrimental effect on the expansion or differentiation of erythroid cultures. This may indicate that the iron-binding affinity of deferiprone is just right to prevent toxicity of free iron, while simultaneously releasing iron to either intracellular proteins or to transferrin molecules. Alternatively, erythroid cells may be protected against high iron levels because of the expression of anti-oxidant proteins^50^.

The recommended dosage of deferiprone for iron-chelation therapy is 75 mg/kg/day to attain deferiprone plasma levels ranging between 10 and 40 mg/L^51–53^. Differentiation medium contains 21.7 mg/L Def (52 µM Def_3_·Fe^3+^). Assuming an equal distribution of deferiprone in the extra- and intracellular space, and a cell volume of 200 pL (Fig. S1a), 1 unit packed reticulocytes (2 × 10^12^ cells) would contain 87 mg deferiprone. For an average human adult (62 kg), the transfusion of a single unit of packed red blood cells would correspond to 0.14 mg/kg deferiprone, two orders of magnitude lower than the recommended deferiprone dosage in a single day of treatment. Even if it is assumed that all deferiprone in the medium is accumulated inside the cultured reticulocytes, the deferiprone dosage would be 70 mg/kg, still lower than the recommended daily dosage of deferiprone. We expected these values to be lower, as cultured reticulocytes must be washed to remove medium components and pyrenocytes before being used for therapeutic purposes.

The use of deferiprone is not without potential side effects. Neutropenia has been observed for deferiprone treatment of thalassemia patients at much higher dosages (75 mg/kg/day) and an incidence of ~ 2 per 100 patient years^54^. We expect, however, a low risk for side effects after transfusion of cRBCs cultured with iron-loaded deferiprone as iron source. Deferiprone is able to redistribute iron among cells, but half of it still contains iron. The deferiprone load in cRBCs is low, it will rapidly diffuse out of the cRBC, and be eliminated from the body via degradation in the liver and excretion via the kidneys with a half-life of 2–3 h^37^.

We hypothesize that Def_3_·Fe^3+^ may act independent of Tf and the TFR1, but only deletion of TFR1/TFR2 will confirm this. Compared to addition of aTf, both Def_3_·Fe^3+^ and hTf treatment of cultured erythroblasts increased ferritin expression and decreased CD71 (TFR1) surface expression, indicating that Def_3_·Fe^3+^ increased intracellular iron concentration. Recently, modulation of Epo-mediated signaling via transferrin and TFR2 was reported, in which the binding of hTf or mTf_C_ to TFR2 increased Epo sensitivity, enhancing the survival, proliferation and differentiation of erythroid progenitor cells, specially at low Epo concentrations (0.1 U/mL)^13,55^. Notably, Epo-induced STAT5 phosphorylation is reduced in absence of hTF, which is not rescued by Def_3_·Fe^3+^. Thus, supplementation of media with Def_3_·Fe^3+^ instead of hTf may shift the Epo dose–response of erythroblasts. The observation that expansion of erythroblast cultures supplemented with Def_3_·Fe^3+^ only is not as high as expansion in presence of hTf plus Def_3_·Fe^3+^ (ca. 50-fold instead of 100-fold in 8 days) may be due to reduced EpoR signaling.

Phosphorylation levels of eIF2α in cells cultured with Def_3_·Fe^3+^ were increased compared to addition of hTf, but lower than in presence of aTf. eIF2α can be phosphorylated by the heme-regulated eIF2α-kinase (HRI), which is activated under heme-limited conditions in erythroid cells^18^. As hemoglobin levels were maintained in Def_3_·Fe^3+^-treated cells, it is possible that the observed increase in p-eIF2α is due to other mechanisms, such as an increase in intracellular oxidative stress levels due to iron overload.

Def_3_·Fe^3+^ in serum-free expansion medium without hTf supplementation supported erythroblast proliferation during the second stage of our culture system. However, Def_3_·Fe^3+^ was unable to support the establishment of erythroblast-enriched cultures from PBMCs. The addition of suboptimal concentrations of hTf to these cultures allows for limited outgrowth of erythroblasts, which was not altered by the presence of Def_3_·Fe^3+^. Possibly, toxicity by iron overload is causing the lower yield. Once cells were committed to the erythroid lineage, Def_3_·Fe^3+^ could fully substitute hTf supplementation.

It is also possible that in the first days of culture, precursor cells not yet committed to the erythroid lineage may lack the molecular machinery to incorporate iron ions provided by Def_3_·Fe^3+^. It becomes relevant to understand how Def_3_·Fe^3+^ complexes are processed by the cells and their interaction with intracellular iron pools, as the data suggests a differential ability between hematopoietic progenitors and erythroblasts to utilize this source of iron. Of note, these early cultures are small scale and the use of hTf is not yet an important cost factor. Thus, cultures could be started in presence of hTf, and be continued in medium supplemented with Def_3_·Fe^3+^ once an erythroblast-enriched population is established.

Holotransferrin is the main iron source in mammalian cell culture medium, and is typically included in culture media by supplementation with serum. However, serum-containing media have an unclear chemical composition, typically show batch-to-batch variation, and have a risk of contamination; for example, by viruses. Defined serum-free formulations have been developed, and require purified hTf^56^. Use of purified Tf from plasma, or of recombinant Tf from rice or yeast, can potentially introduce contaminants. Def_3_·Fe^3+^, however, is chemically produced, which can be an advantage with respect to purity in the production of chemically-defined protein-free medium.

Migliaccio et al.^5^ proposed the first serum-free medium formulation for erythroid culture, and showed that transferrin levels have a direct impact on the yields of erythroid progenitor cells. Erythroblast expansion and differentiation can be achieved using 300 and 1000 µg/mL hTf, respectively^22^. Olivier et al. developed chemically defined culture media for cRBC production in which supplemented Fe^3+^-EDTA reloads the used Tf, allowing for lower Tf concentrations^29^. Nevertheless, 50 µg/mL Tf are still needed in this strategy to reach growth and enucleation levels like those of cultures using Tf-only medium. The results of our study indicate that although transferrin supplementation of serum-free medium is currently essential for the establishment of erythroid cultures from PBMCs, it can be fully replaced by Def_3_·Fe^3+^ supplementation during erythroblast expansion, differentiation and maturation. In the medium described in Heshusius et al.^22^, holotransferrin constitute 65% of the costs of the differentiation medium, including the cost of Epo. Iron-loaded deferiprone at the concentration described in our manuscript (52 μmol/L Def_3_·Fe^3+^) is at least 400-fold less expensive than recombinant transferrin and thus reduces the medium price by 64%^57^. At 50 μg/ml as proposed by Olivier et al.^29^, hTf would constitute 8% of the costs, still considerably more than ~ 0.5% of the costs using only Def_3_·Fe^3+^. Although hTf concentrations are significantly lower in culture media for other primary cells and for cell lines typically used in biopharmaceutical processes (e.g. hybridoma, CHO, Vero; ranging between 5 and 100 µg/mL), its replacement with Def_3_·Fe^3+^ may be a significant cost-saver at an industrial production scale. Furthermore, replacing transferrin by a small iron chelator could help towards protein-free culture medium formulations, which could speed up translations of stem cell cultures to conditions compliant with good manufacturing practices (GMP).

## Methods

### Cell culture

Donor-derived buffy coats are a Sanquin not-for-transfusion product from which PBMCs are purified by density centrifugation. Informed written consent was given by donors to give approval for the use of waste material for research purposes, and was checked by Sanquin’s NVT Committee (approval file number NVT0258; 2012) in accordance with the Declaration of Helsinki and the Sanquin Ethical Advisory Board. Erythroid cells were cultured from human peripheral blood mononuclear cells (PBMCs) in serum-free medium as previously described^22^ with minor modifications to Cellquin medium, which lacked nucleosides, and contained a defined lipid mix (Sigma-Aldrich; USA; 1:1000) replacing cholesterol, oleic acid, and L-α-phosphatidylcholine. Transferrin concentrations varied as indicated. hTf (Sanquin; Netherlands) or aTf (Sigma-Aldrich) were added as indicated. In the first phase, erythroblast cultures are established from PBMC in presence of erythropoietin (Epo, 1 U/mL; EPREX®, Janssen-Cilag, Netherlands), hSCF (100 ng/mL, produced in HEK293T cells), dexamethasone (1 µmol/L; Sigma-Aldrich), and IL-3 (1 ng/mL first day only; Stemcell Technologies; Canada). From day 6 erythroblast cultures were expanded and the cell density was maintained between 0.7–2 × 10^6^ cells/mL (CASY Model TCC; OLS OMNI Life Science; Germany). To induce differentiation, cells were washed and reseeded at 1–2 × 10^6^ cells/mL in presence of Epo (5 U/mL), 5% Omniplasma (Octapharma GmbH; Germany), heparin (5 U/mL; LEO Pharma A/S; Denmark). During this differentiation phase the cells undergo 3–4 divisions before they mature to enucleated reticulocytes. AML-derived cell lines MOLM13^58^, NB4^59^, EOL1^60^, K562^61^, HL60^62^ and ML2^63^ were seeded at a concentration of 0.3 × 10^6^ cells/mL in Cellquin, supplemented with hTf and Def_3_·Fe^3+^ as indicated.

### Iron chelators

Deferiprone (Def; Sigma-Aldrich), deferoxamine mesylate (DFOA; Sigma-Aldrich), deferasirox (DFO; Sigma-Aldrich) and hinokitiol (HINO; Sigma-Aldrich) were dissolved as indicated by the manufacturer and mixed at stoichiometric ratio with an iron(III) chloride solution (Sigma-Aldrich; 16 h, 20 °C), to obtain a concentration of 26 mmol/L chelated Fe^3+^ (Def_3_·Fe^3+^, DFOA·Fe^3+^, DFO_2_·Fe^3+^, HINO_3_·Fe^3+^), equivalent to the iron content of 1000 mg/mL hTf.

### Flow cytometry

Cells were stained in HEPES buffer + 0.5% BSA (25–30 min, 4 °C), measured using a BD FACSCanto™ II flow cytometer (BD Biosciences), gated against specific isotypes, and analyzed using FlowJo™ (version 10.3; USA). Antibodies or reagents used were: (i) CD235a-PE (1:2500 dilution; OriGene cat#DM066R), CD49d-BV421 (1:100 dilution; BD-Biosciences cat#565,277), DRAQ7 (live/dead stain; 1:200 dilution; ThermoFischer Scientific cat#D15106); (ii) CD235a-PE (1:2500 dilution; OriGene cat#DM066R), CD71-APC (1:200 dilution; Miltenyi cat#130-d099-219); (iii) CD71-VioBlue (1:200 dilution; Miltenyi cat#130-101-631); (iv) DRAQ5 (nuclear stain; 1:2500 dilution; abcam cat#ab108410); (v) PI (live/dead stain; 1:2000 dilution; Invitrogen cat#P3566).

### Western blots

Iron saturation of transferrin was measured on 6% TBE-urea gels as previously described^64^. Gels were stained with InstantBlue Coomassie protein stain (abcam).

For the determination of the expression level of proteins involved in iron metabolism, whole cell lysates harvested during differentiation were prepared in RIPA buffer (10 min, 4 °C). For Epo induction experiments, erythroblasts cultured in expansion medium supplemented with 300 µg hTf or 52 µM Def_3_·Fe^3+^ and Epo (2 U/mL) were washed twice in PBS and reseeded in expansion medium with hTf and Def_3_·Fe^3+^ but without Epo. After 3 h, cells were stimulated with Epo as indicated for 20 min at 37 °C, washed in ice-cold PBS and harvested in RIPA buffer.

Protein concentration was determined by colorimetry (DC™ Protein Assay; Bio-Rad; USA). Lysates were diluted 1:4 with Laemmli sample buffer (Bio-Rad), incubated for 95 °C (5 min), subjected to SDS–polyacrylamide gel electrophoresis (4–20% Criterion™ Tris–HCl Protein Gel, Bio-Rad), transferred to nitrocellulose membranes (iBlot2 system; ThermoFischer Scientific), and stained. Membranes were cut after protein transfer to reduce the volume of antibodies needed for staining. Primary antibodies included actin (Sigma cat#A3853), transferrin receptor (Merck cat#SAB4200398), ferritin (abcam cat#ab75973), p-eIF2 (CellSignaling cat#3597L), total eIF2 (CellSignaling cat#9722S), p-STAT5 (Millipore; USA; cat#05–495), and total STAT5 (Santa Cruz; USA; cat#SC-835). Western blots were analyzed using GelAnalyzer 19.1. Original uncropped blot images are available as Supplementary Data, and are also deposited in the publicly available data repository Zenodo under the public link https://doi.org/10.5281/zenodo.6350135.

### Hemoglobin quantification and oxygenation

Hemoglobin content was determined using *o*-phenylenediamine as described^65^. Intracellular hemoglobin concentration was calculated using the mean of technical triplicates and the cell volume (CASY Model TCC). Oxygen association/dissociation curves for RBCs were determined by continuous dual wavelength spectrophotometric measurement with a HEMOX Analyzer (TCS Scientific Corp.; USA), using RBCs that were washed and resuspended at 20 × 10^6^ cells/mL in analysis buffer (PBS + 0.5% BSA + 0.005% Y-30 antifoam). P_50_ values were calculated as the oxygen partial pressure that leads to a 50% saturation of hemoglobin.

### Statistical analysis

Statistical analyses were performed using the two-tailed two sample Student’s *t*-test. For comparison of fold change (FC) data, log(FC) values were first calculated and then used for the statistical test. When indicated, *p*-values were corrected for multiple comparisons with the Holm-Bonferroni method. All data in figures is displayed as mean ± the standard deviation of the measurements. The number of replicates is n ≥ 3 for all experiments. Significance is expressed as: ns for not significant differences, * for *p* < 0.05, ** for *p* < 0.01, *** for *p* < 0.001).

## Supplementary Information

Supplementary Information.

## Data Availability

Original blot images corresponding to the data presented in Figs. 3 and 4 are deposited in the publicly available data repository Zenodo under the public link https://doi.org/10.5281/zenodo.6350135. For original data, please contact m.vonlindern@sanquin.nl.
