# Supplementary material for: Iron-loaded deferiprone can support full hemoglobinization of cultured red blood cells
Source: Sci Rep. 2023 Apr 28;13:6960. doi: 10.1038/s41598-023-32706-1 (PMC10147612; doi:10.1038/s41598-023-32706-1)
Supplement: Supplementary file 1 — Supplementary Information. [file 41598_2023_32706_MOESM1_ESM.pdf]

Iron-loaded deferiprone can support full hemoglobinization of cultured red blood cells

Supplementary Information

Joan Sebastián Gallego-Murillo[1,2,+], Nurcan Yağcı[1], Eduardo Machado Pinho[1,3], Aljoscha Wahl[2,‡], Emile van den Akker[1], Marieke von Lindern[1*]

[1] Department of Hematopoiesis, Sanquin Research and Landsteiner Laboratory, Amsterdam University Medical Center (UMC), Amsterdam, The Netherlands

[2] Department of Biotechnology, Faculty of Applied Sciences, Delft University of Technology, Delft, The Netherlands

[3] Department of Bioengineering, Faculty of Engineering, University of Porto, Porto, Portugal

[+] current address: Meatable, Alexander Fleminglaan 1, 2613AX Delft, The Netherlands;

[‡] current address: Lehrstuhl Für Bioverfahrenstechnik, Friedrich-Alexander Universität Erlangen-Nürnberg, Paul-Gordan-Str. 3, 91052 Erlangen, Germany.

* Corresponding author

E-mail: m.vonlindern@sanquin.nl

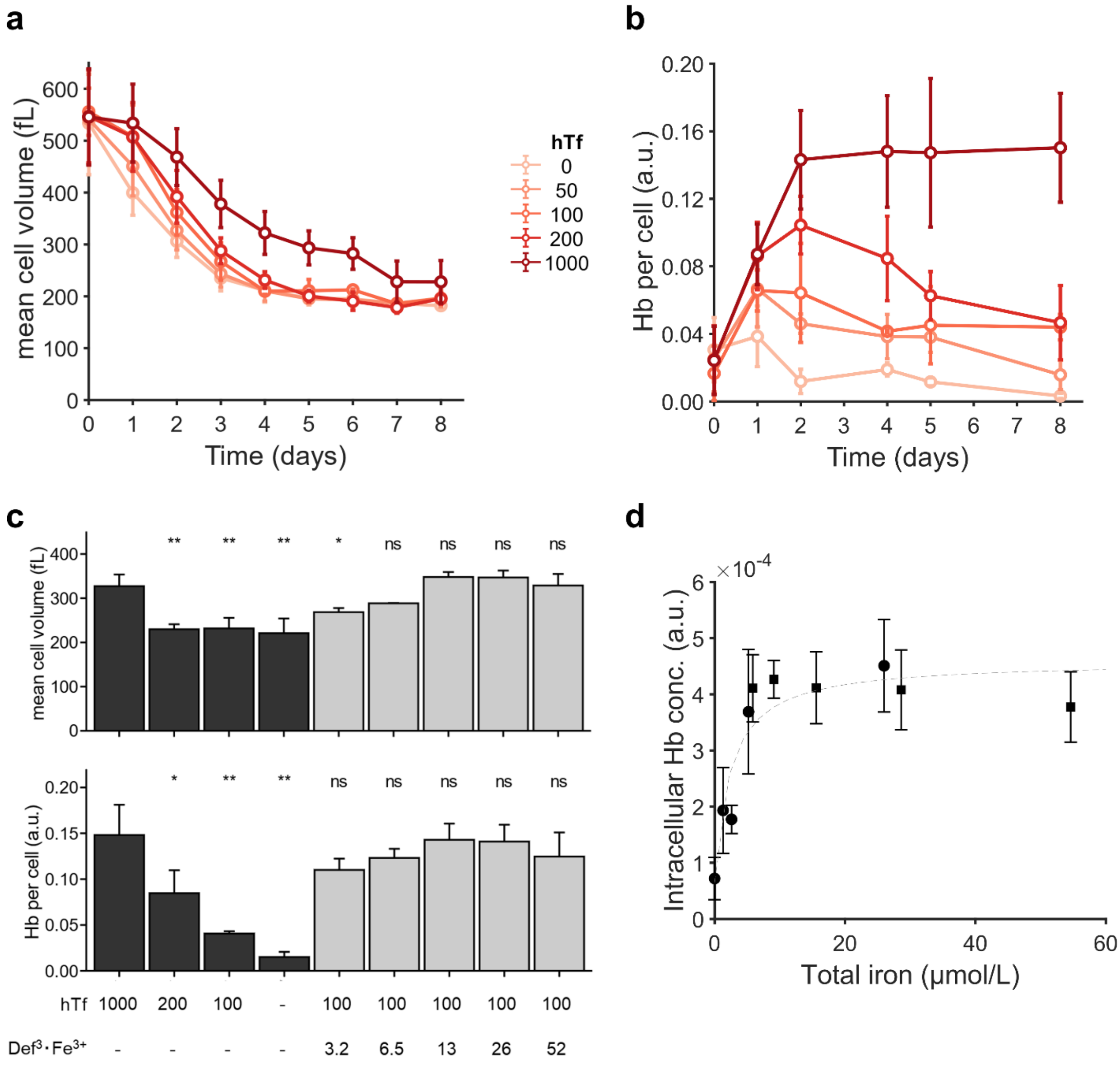

Supplementary Figure S1. Intracellular hemoglobin concentration of differentiating erythroblasts using holotransferrin and iron-loaded deferiprone as iron source. Erythroblasts were expanded from PBMCs for 10-12 days, and subsequently seeded in differentiation medium at a starting cell concentration of 1.5 – 2.0 million cells. **(a)** Cells were seeded with decreasing holotransferrin concentrations (1000, 200, 100, 50 and 0 µg/mL). Evolution of mean cell diameter is depicted. **(b)** Hemoglobin cell content (i.e. mass of hemoglobin per cell; displayed as arbitrary

units = absorbance at 420 nm per million cells) was determined at indicated days. The effect of deferiprone supplementation with sub-optimal hTf concentrations on erythroblast cell volume **(c)** and hemoglobin content **(c,d)** in differentiation cultures was also evaluated. For this, erythroblast expanded from PBMCs were seeded in medium using hTf as sole iron source (dark grey; ●), or in hTf-limited conditions (100 µg/mL hTf) in the presence of $Def_3 \cdot Fe^{3+}$ in concentrations ranging between 3.2 and 52 µmol/L (light grey; ■). Intracellular hemoglobin concentration was calculated using the cell volume and Hb per cell values (day 4 of treatment), and is depicted in arbitrary units (a.u.) of absorbance per fL of cell volume. Mean ± SD (error bars; n≥3). Significance is shown for the comparison with 1000 µg/mL hTf (unpaired two-tailed two-sample Student's *t*-test).

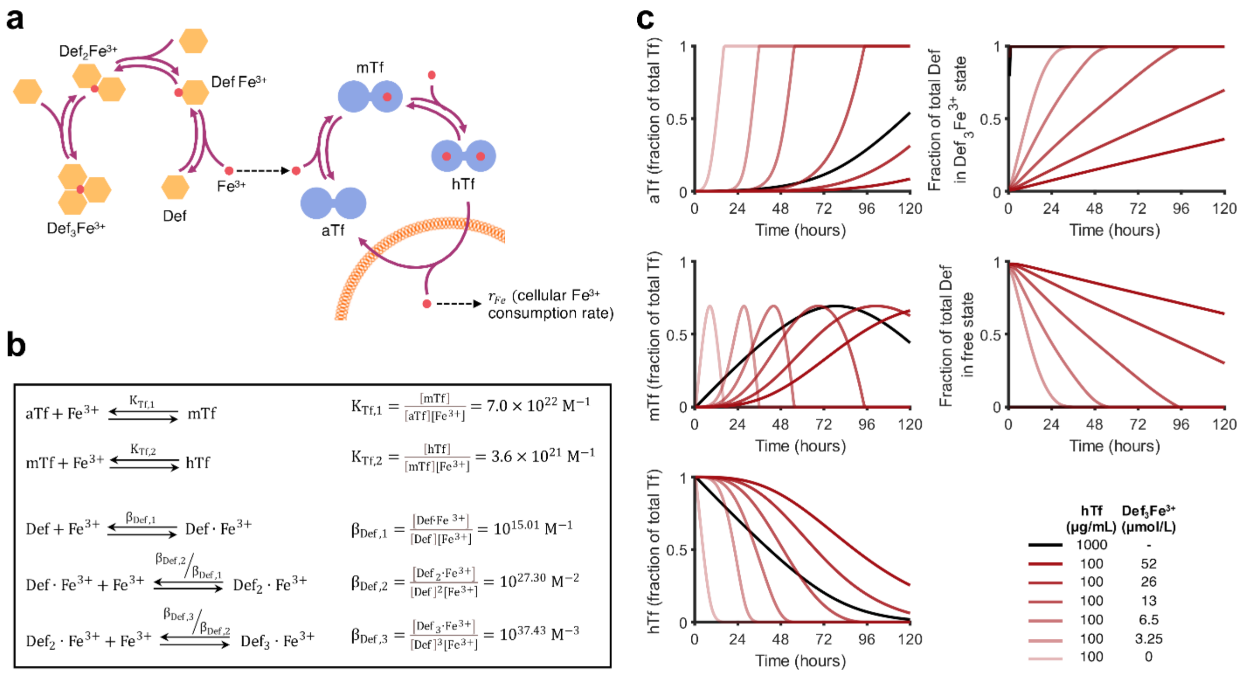

Supplementary Figure S2. Modeling of iron saturation in transferrin and deferiprone solutions. (a) Iron shuttling mathematical model proposed for the reloading of apotransferrin in culture using iron-loaded deferiprone. Leaching of Fe^{3+} ions from deferiprone or transferrin was the only mechanism considered for the transfer of iron between the two chelators. **(b)** Concentrations of transferrin and deferiprone species were calculated assuming equilibria at culture conditions (pH = 7.4, 3.5 mmol/L HCO_3^-). For transferrin, the association equilibrium constants of 7.0×10^{22} L/mol and 3.6×10^{21} L/mol for the binding of the first and second Fe^{3+} ion were assumed, respectively [36]. No difference in the association of iron to the N- and C-lobes of transferrin was considered. For deferiprone, the global stability constants (log β) for the $Def{\cdot}Fe^{3+}$, $Def_2{\cdot}Fe^{3+}$ and $Def_3{\cdot}Fe^{3+}$ complexes were assumed to be 15.01, 27.30 and 37.43, respectively [32]. A constant net average iron uptake rate (inflow + iron export from the cells) of 1.7×10^{-7} mol Fe^{3+}/L·h was assumed, corresponding to the production of 10×10^6 hemoglobinized cells per mL of culture (1 cell = 300 million Hb molecules) in 4 days, for simplicity purposes. It was assumed that iron

association and dissociation kinetics with Tf and Def dominate iron concentration in the extracellular space, and that this is faster than the net utilization rate of iron in the intracellular space. Calculation of transferrin and deferiprone concentrations for each timepoint was performed solving the system of nonlinear chemical equilibrium equations with MATLAB ver. R2019b. **(c)** Calculated time profiles for the concentrations of the different transferrin and deferiprone species for cultures with either 1000 or 100 μg/mL of hTf, in presence or absence of $Def_3{\cdot}Fe^{3+}$. Under low hTf conditions, supplementation with $Def_3{\cdot}Fe^{3+}$ leads to a delay on the depletion of hTf.

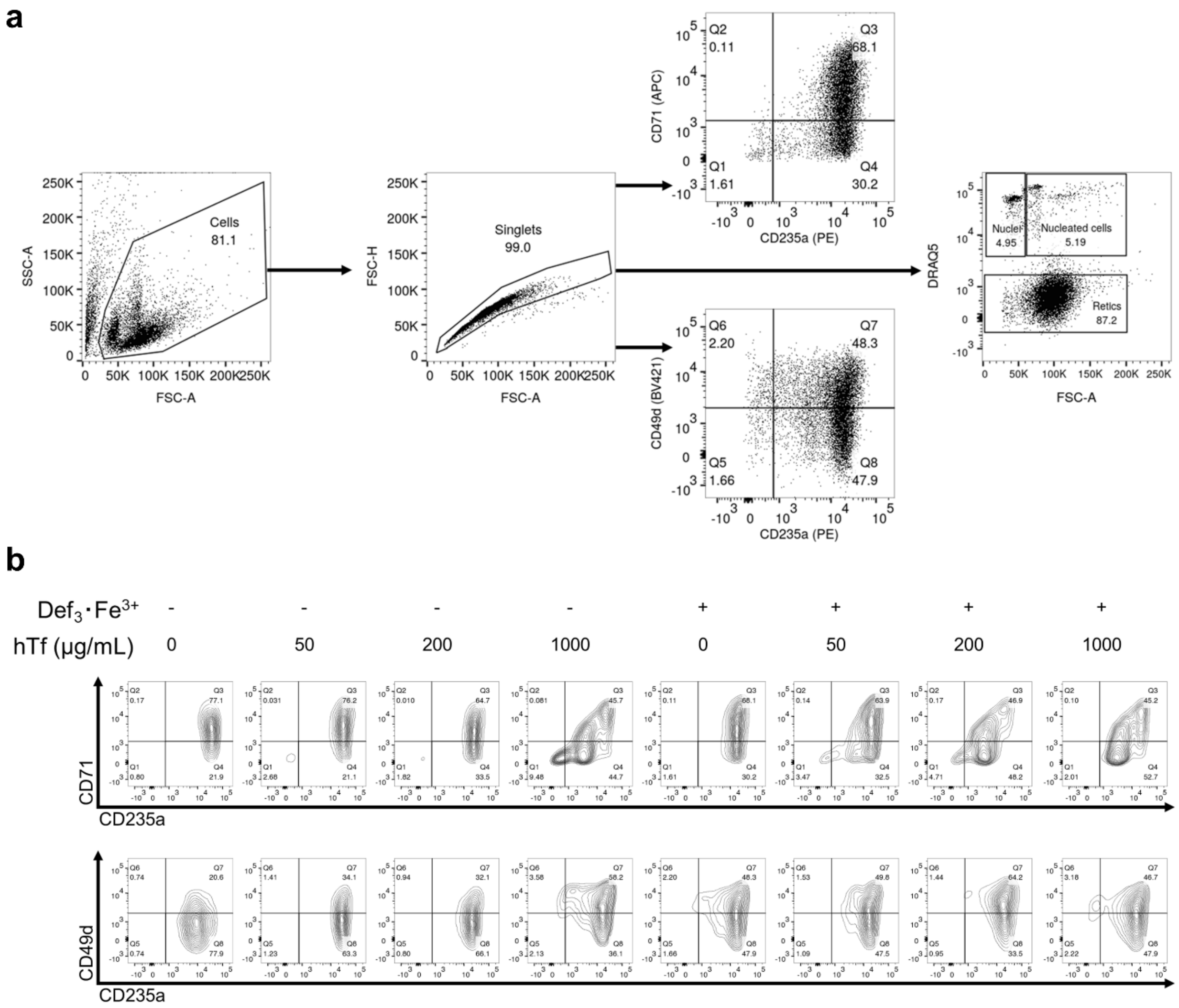

Supplementary Figure S3. Expression of erythroid cell surface markers in differentiation cultures supplemented with deferiprone. Erythroblasts were differentiated for 10 days in medium at different hTf concentrations, in the presence or absence of $Def_3 \cdot Fe^{3+}$ (52 µmol/L). **(a)** Gating strategy to evaluate the differentiation level of cultured erythroblasts. Cells were gated (FSC/SSC), followed by gating of single cells (FSC-A/FSC-H). Cells are depicted in a CD235a/CD71 or a CD235a/CD49d dot plot. To evaluate enucleation levels, erythroblasts ($DRAQ5^+$ FSC^{high}), pyrenocytes (extruded nuclei; $DRAQ5^+$ FSC^{low}) and reticulocytes ($DRAQ5^-$)

were gated. **(b)** Representative density plots indicating the expression of the cell surface markers CD71, CD235 and CD49d after 10 days of culture.

a.1

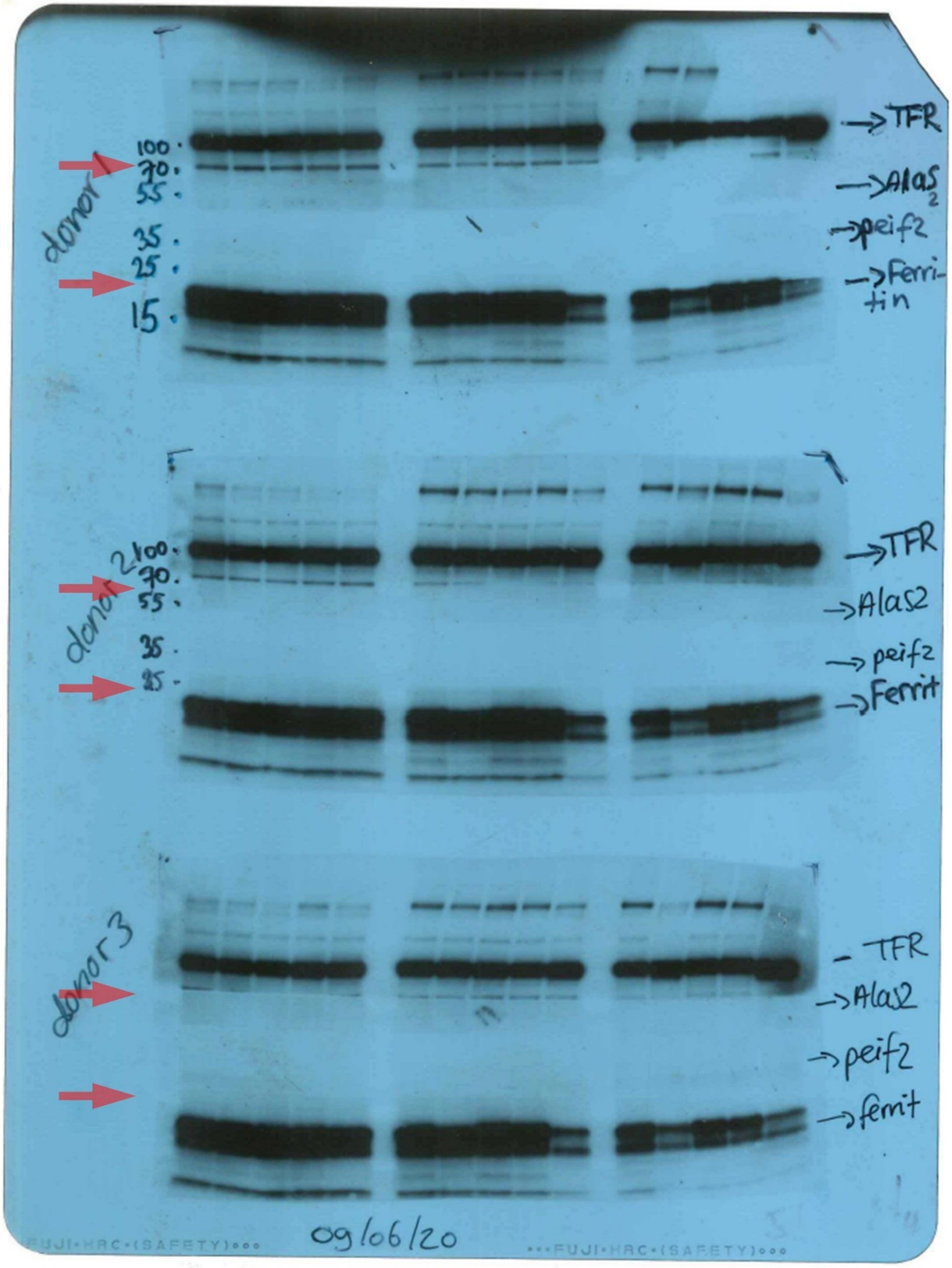

(continues in following page)

a.2.i

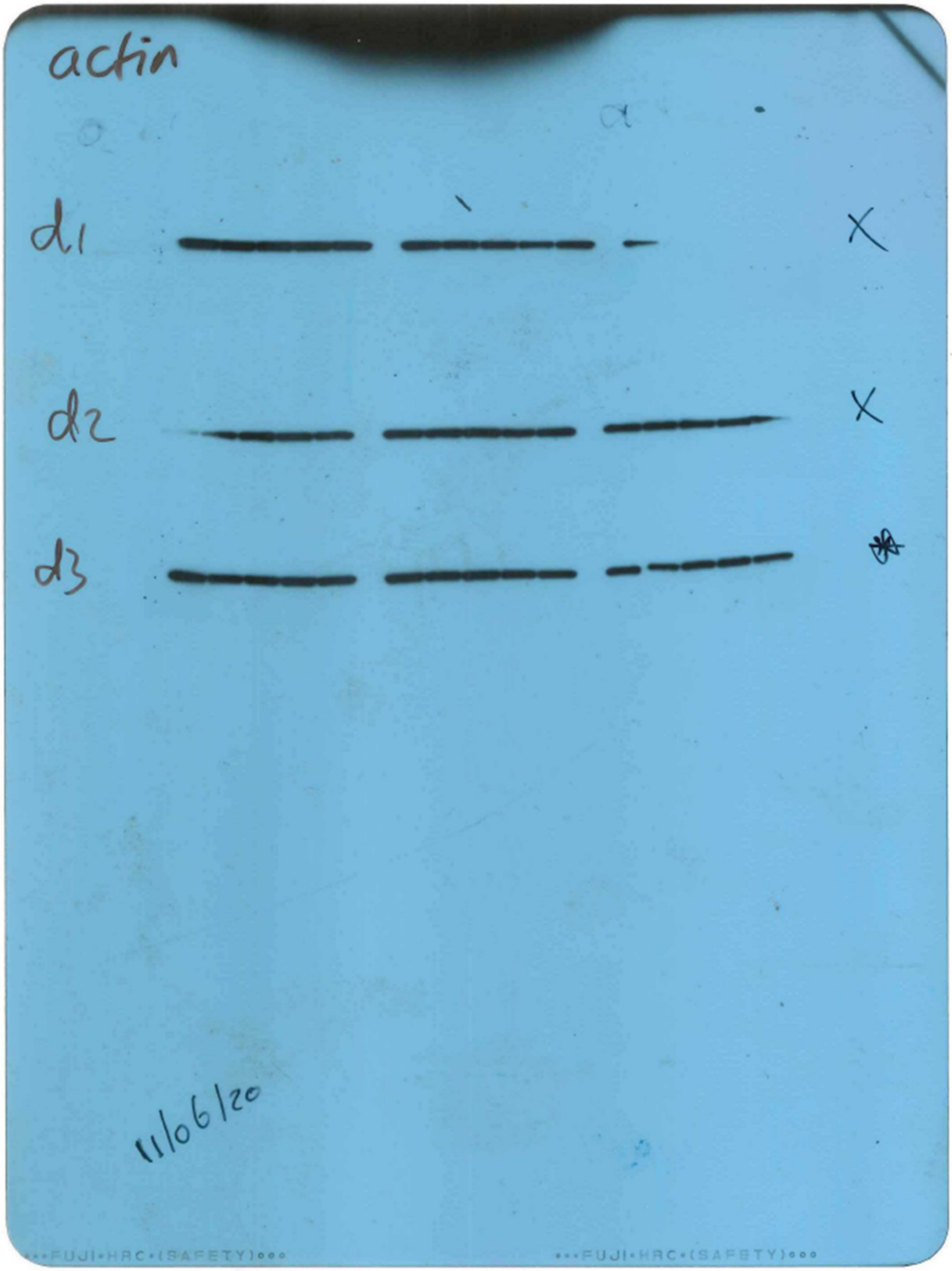

(continues in following page)

a.2.ii

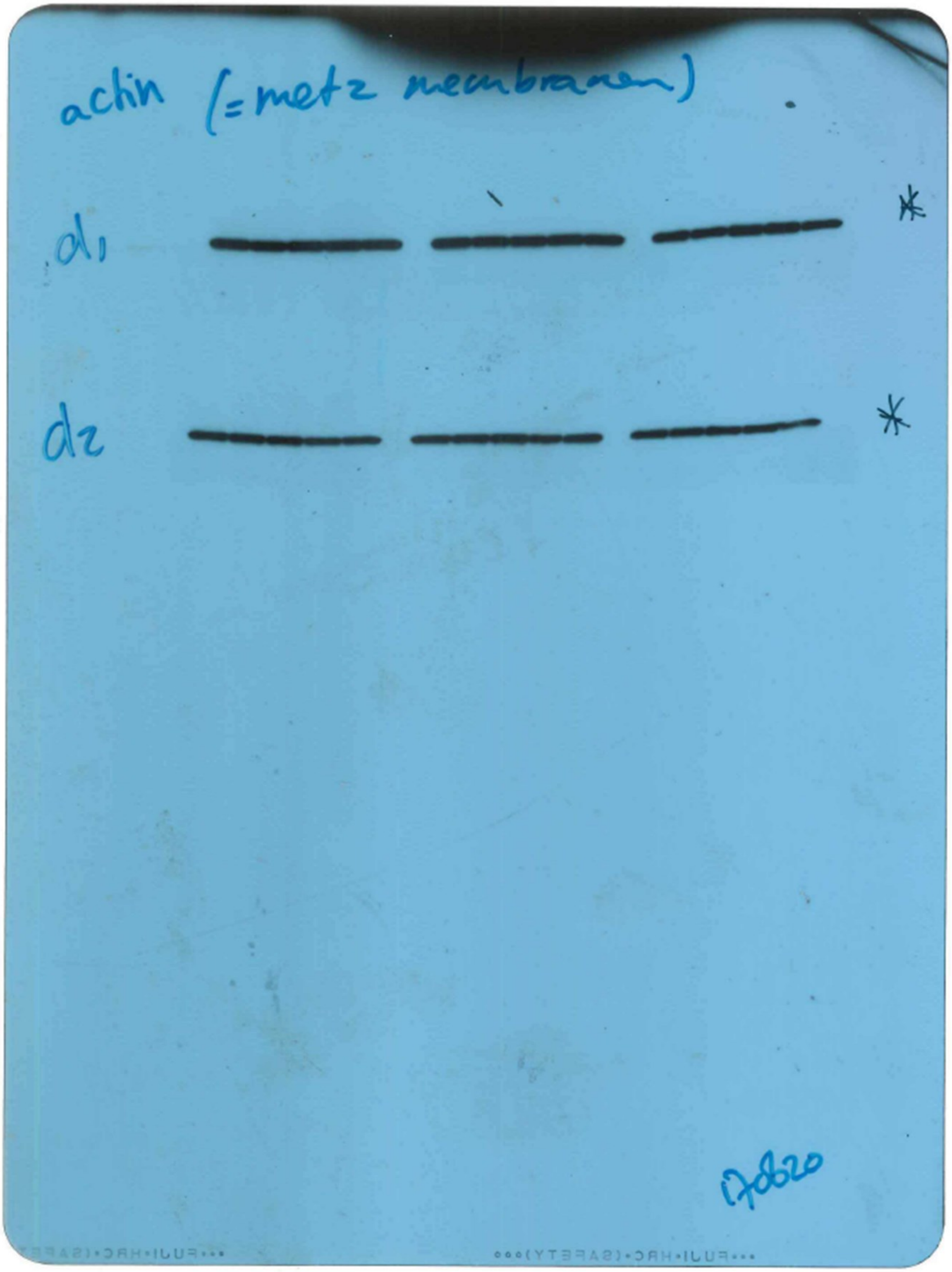

(continues in following page)

a.3

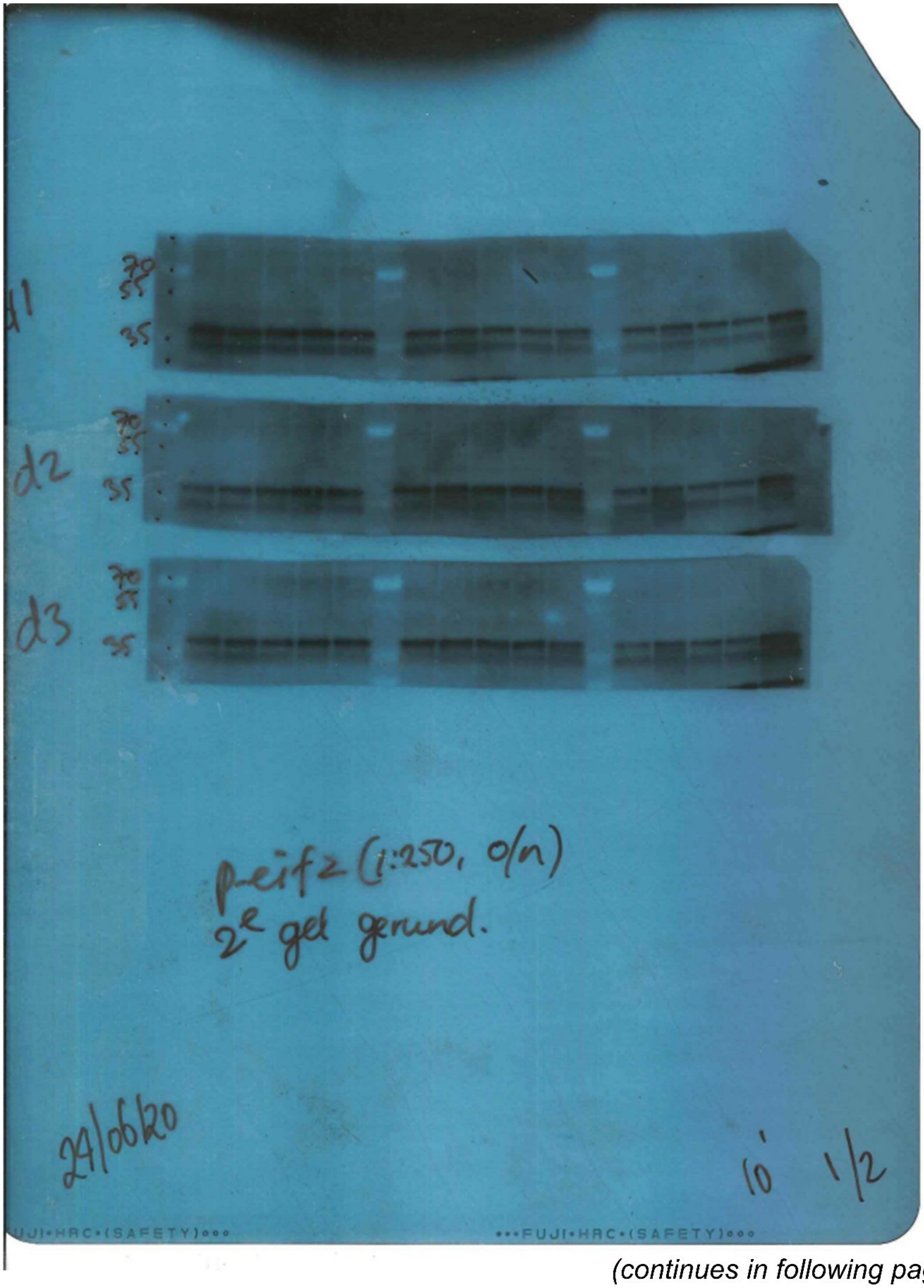

(continues in following page)

70

a.4

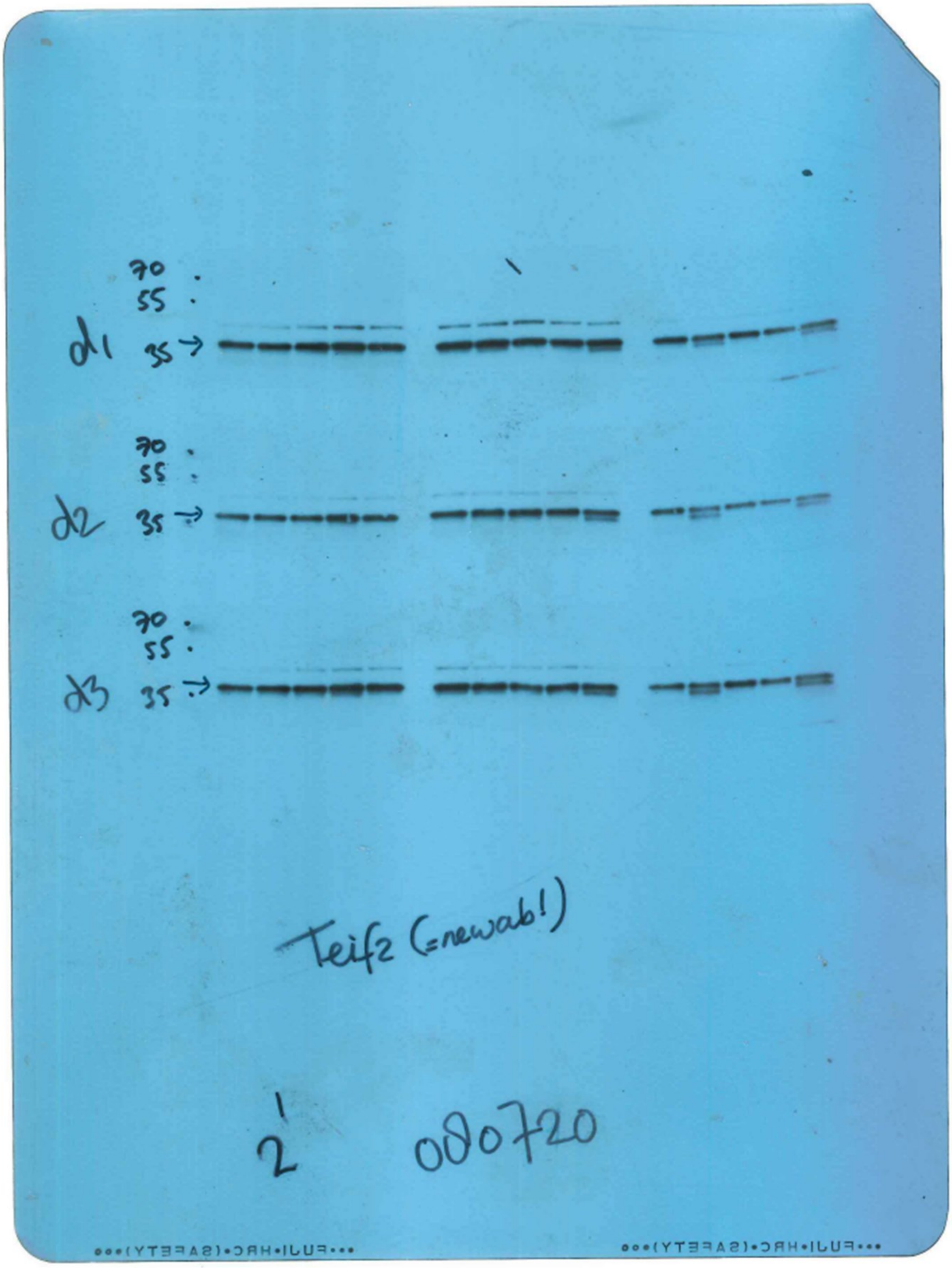

(continues in following page)

a.5

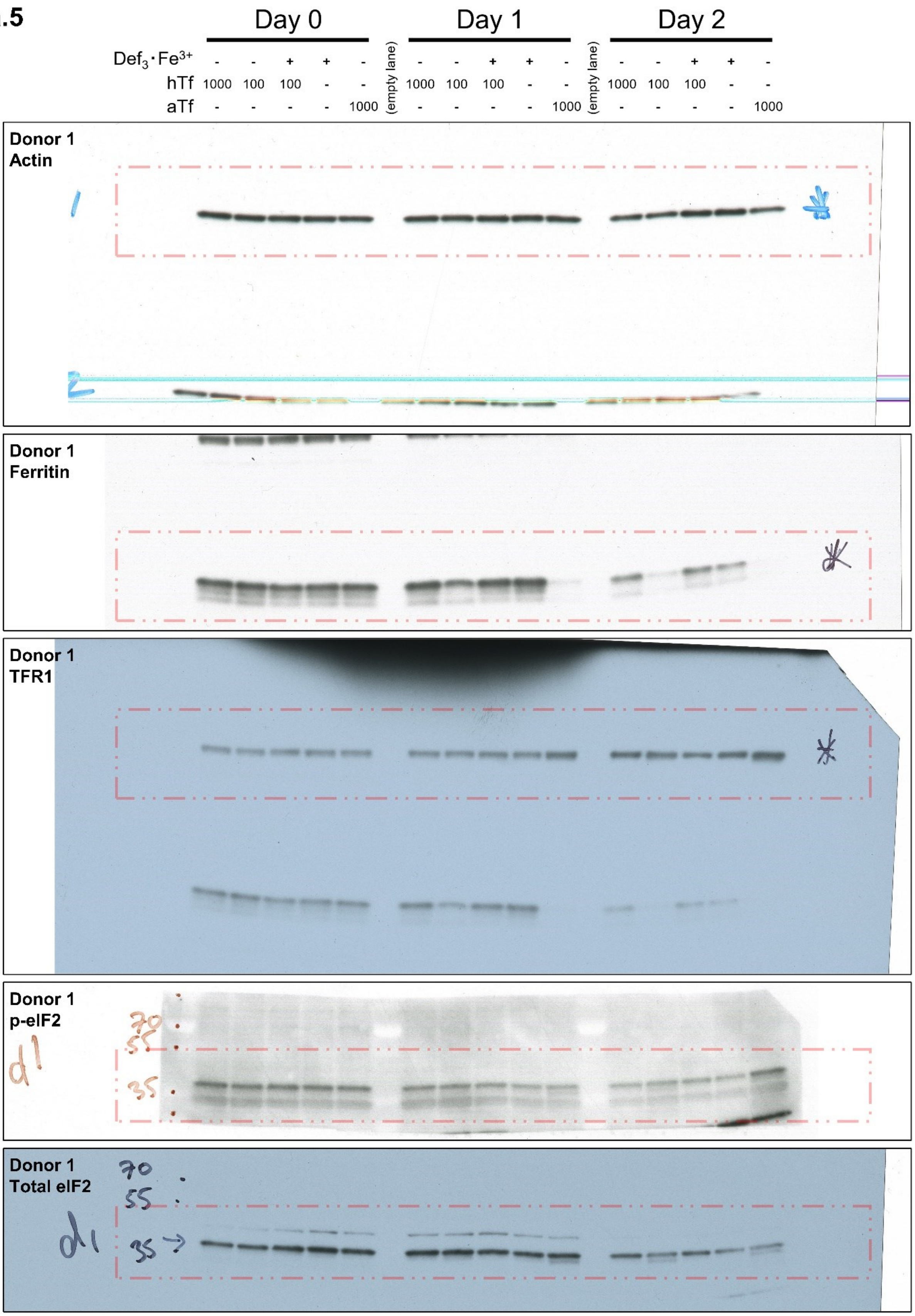

(continues in following page)

a.6

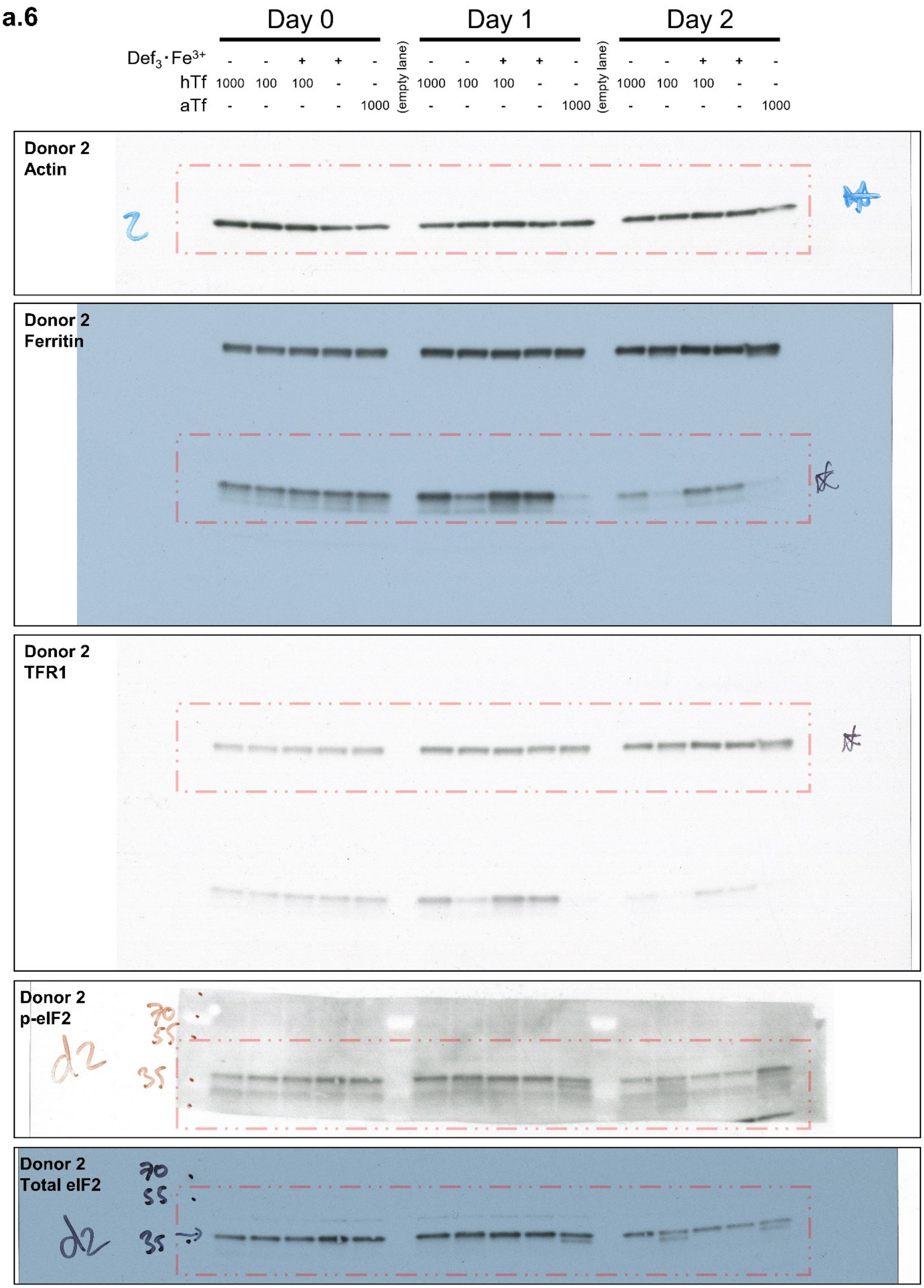

(continues in following page)

73

a.7

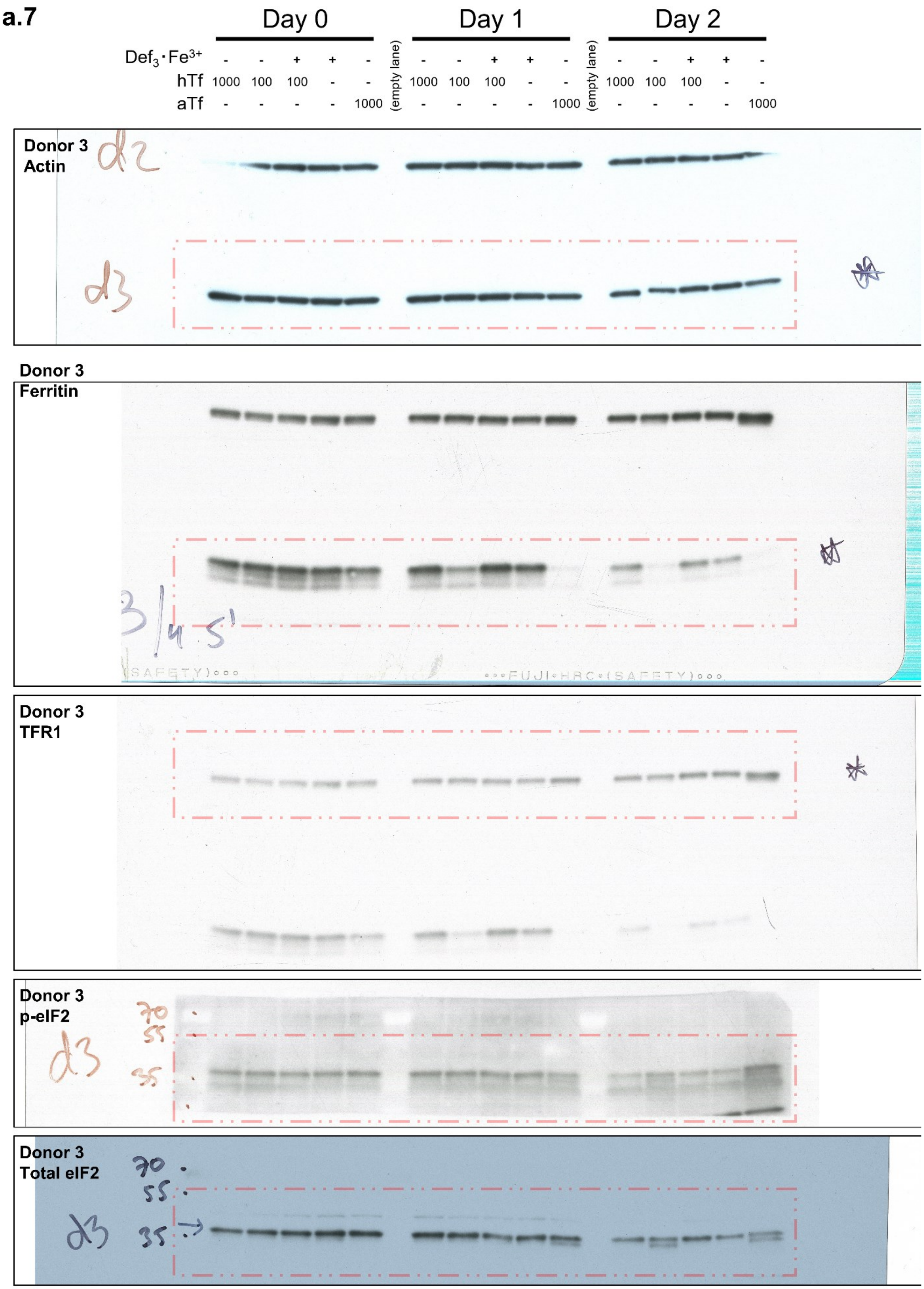

Day 0
Day 1
Day 2
Def3·Fe3+ - - + + - (empty lane) - - + + - (empty lane) - - + + -
hTf 1000 100 100 - - 1000 100 100 - - 1000 100 100 - -
aTf - - - - 1000 - - - - 1000 - - - - 1000
Donor 3
Actin
Donor 3
Ferritin
Donor 3
TFR1
Donor 3
p-eIF2
Donor 3
Total eIF2

Supplementary Figure S4. Raw images of Western blot membranes used for the quantification of transferrin receptor, ferritin, phosphorylated and total eIF2 of Figure 3. Blots were cut in fragments to stain for distinct proteins. **(a1)** Transferrin receptor, ferritin and actin were probed on the same blot. This blot was cut in 4 parts. The top fragment (above the red arrows) was probed for the transferrin receptor, the lowest part (below the red arrow) was probed for ferritin. (Middle parts were unsuccessful). All parts were placed together following ECL staining, and the reconstructed blots were exposed for various times to x-ray films. Size markers are indicated. **(a2)** The third membrane fragment **(a2i)** or the second plus third membrane fragment **(a2ii;** counted from the top) were probed for actin**. (a3-4)** Phospho-eIF2 **(a3)** and the total eIF2 **(a4)** were probed on blots made from an independent gel, but with the same samples run on gels shown in (a1) and (a2). Total eIF2 is the loading control of phospho-eIF2. Distinct exposures of the same blot membranes displayed in panels (a1) and (a2) were scanned and used for the quantitative analysis of Figure 3 (see **a5**, donor1; **a6**, donor2; **a7**, donor3), with dotted red box indicating the regions of interest. All images were obtained scanning exposed x-ray films (Epson V500 scanner, full color, 1200 dpi, bit depth=24). Individual images are available in the publicly available data repository Zenodo under the public link https://doi.org/10.5281/zenodo.6350135.

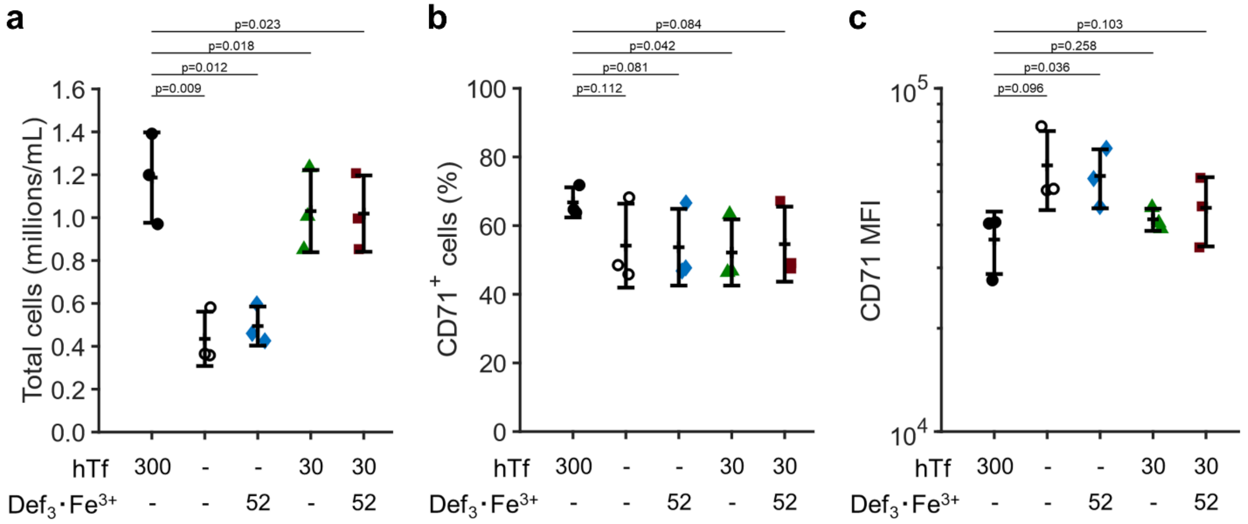

Supplementary Figure S5. Cell yields and expression of transferrin receptor TFR1 (CD71) on erythroid cultures stablished under deferiprone supplementation. PBMCs were cultured in expansion medium without iron, or supplemented with holotransferrin (30 µg/mL or 300 µg/mL) and $Def_3 \cdot Fe^{3+}$ (52 µmol/L), as indicated. After 6 days of culture, the typical time for the establishment of erythroid cultures, total cell concentration was measured **(a)**. Percentage of $CD71^+$ cells **(b)** and the mean fluorescence intensity of CD71 **(c)** were determined by flow cytometry. Cultures were kept for 9 days more, as shown in Fig. 4b. Mean ± SD (error bar; n=3). Significance is shown for the comparison with the 300 µg/mL hTf condition (paired two-tailed two-sample Student's *t*-test).

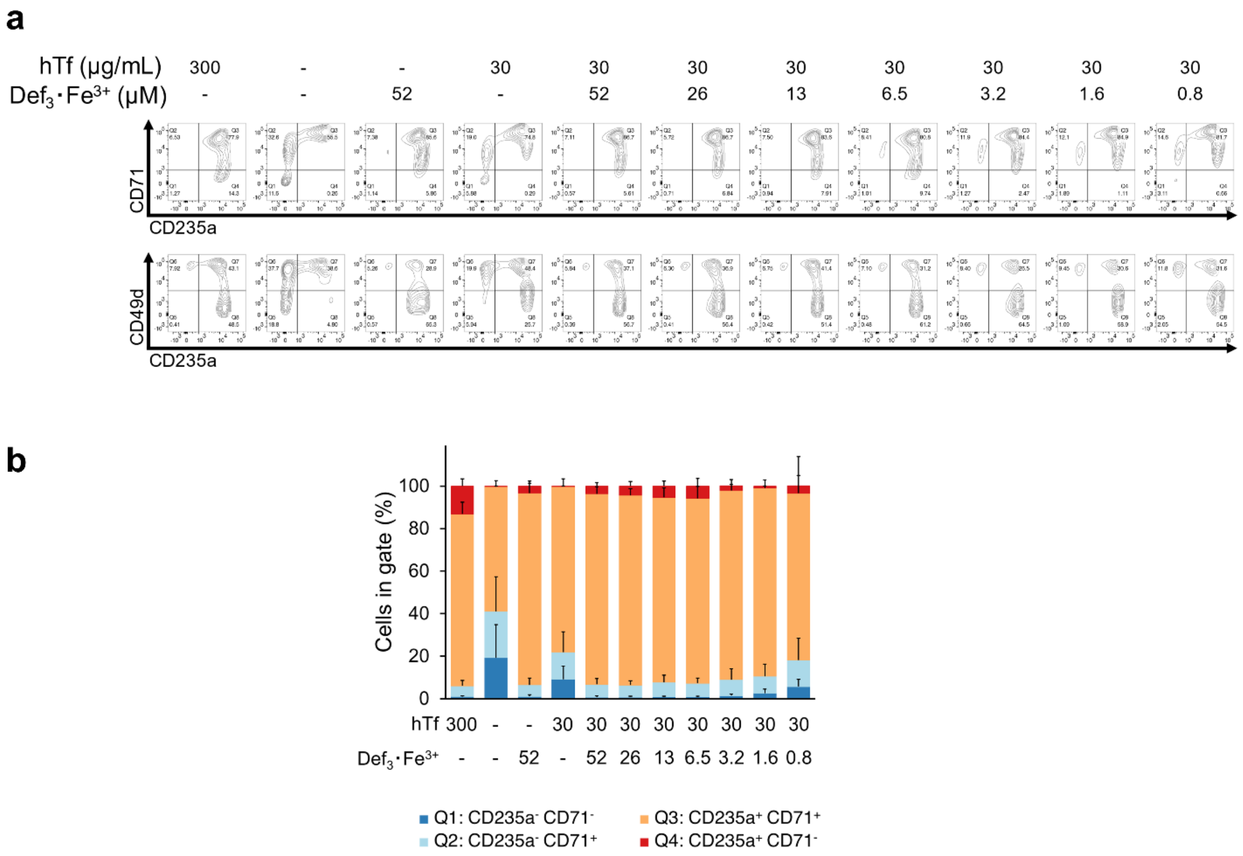

Supplementary Figure S6. Expression of erythroid cell surface markers in erythroblasts expanded in medium supplemented with deferiprone. PBMCs were cultured in our original expansion medium (300 µg/mL hTf) for 7 days until an erythroblast-enriched culture was obtained, followed by further culturing using 300, 30 or 0 µg/mL hTf in the presence of $Def_3 \cdot Fe^{3+}$ (0.8 – 52 µmol/L $Def_3 \cdot Fe^{3+}$). **(a)** Representative density plots indicating the expression of the cell surface markers CD71, CD235 and CD49d curves after 8 days of treatment (culture age = 15 days since PBMC isolation). **(b)** Relative cell numbers per quadrant (CD235a vs. CD71) were calculated for 3 different donors. Data in the bar plot is displayed as mean ± SD (error bars; n=3).

a

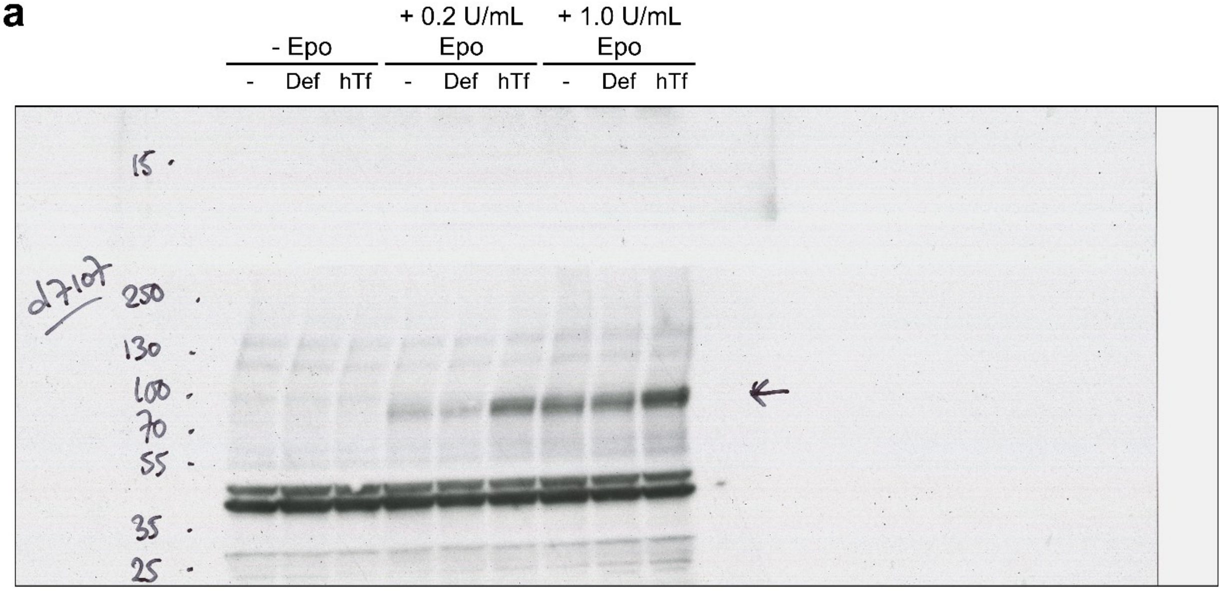

b

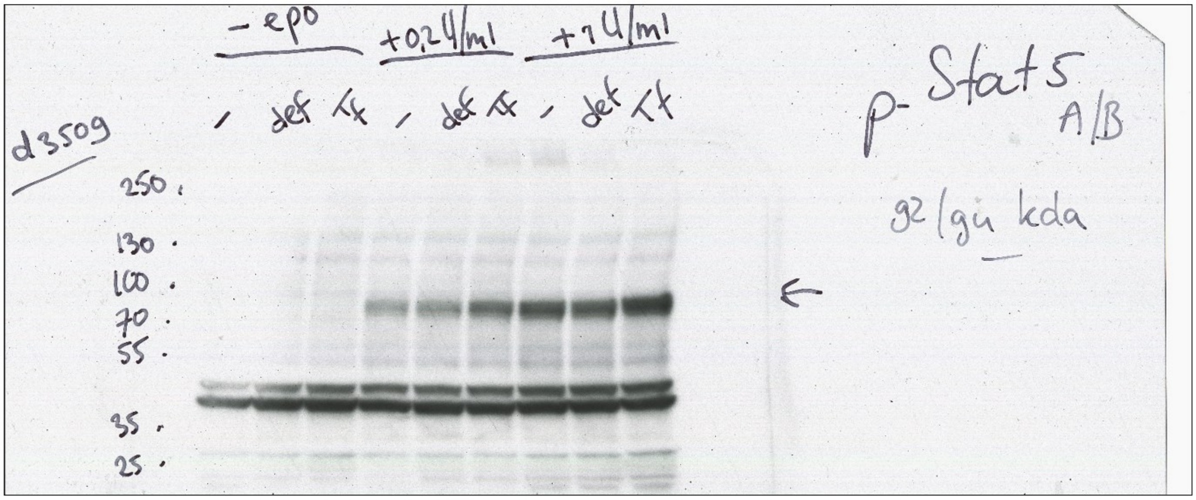

(continues in following page)

111

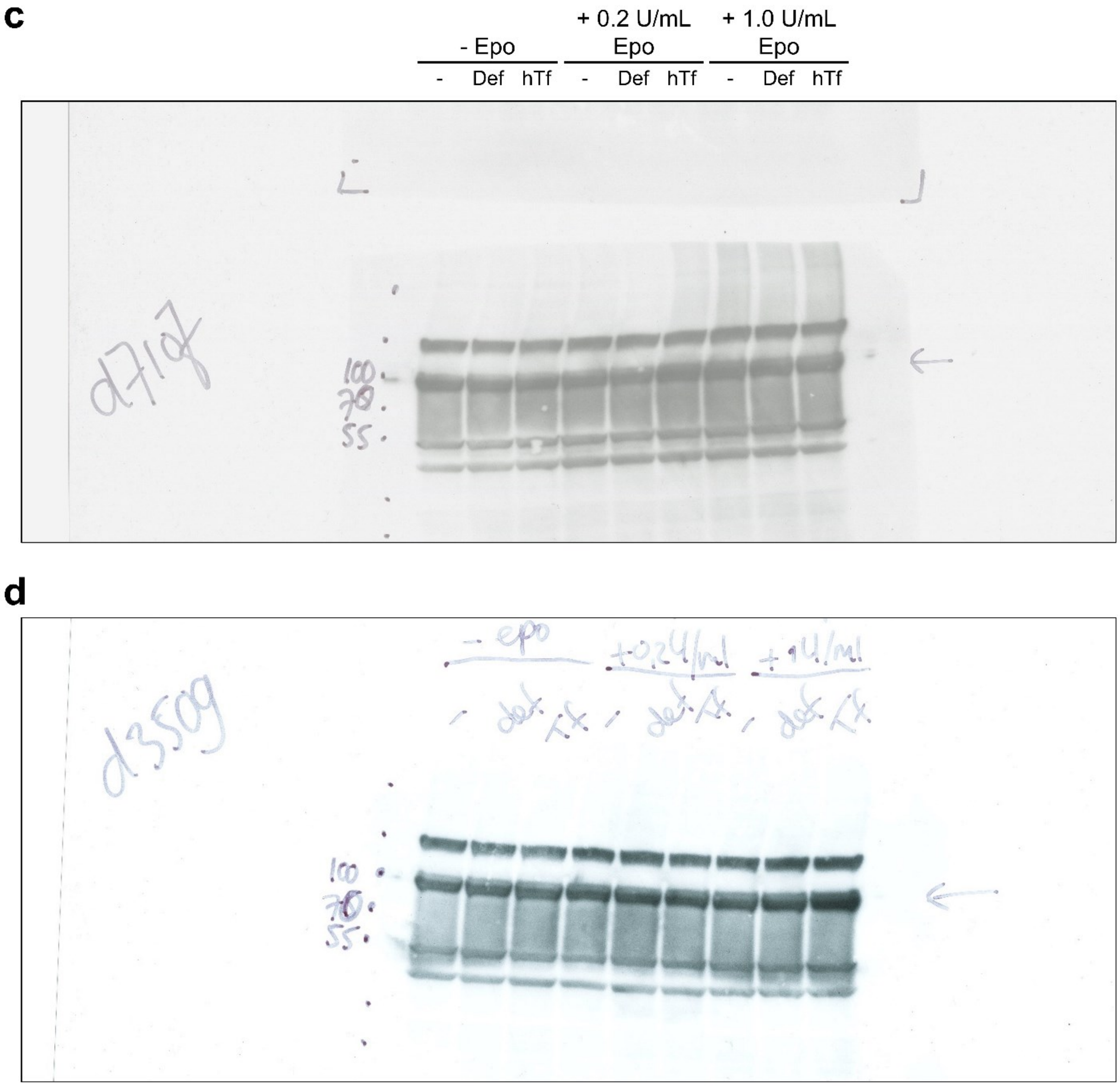

Supplementary Figure S7. Raw images of Western blot membranes used for quantification of phosphorylated and total STAT5 of Figure 4g. Blots were stained with anti-phospho-STAT5 **(a, b)**, and next with total STAT5 antibodies **(c, d)**. Size markers are indicated. Following ECL staining, membranes were exposed for various times to x-ray films, which were subsequently scanned in an Epson V500 scanner (full color, 1200 dpi, bit depth = 24).

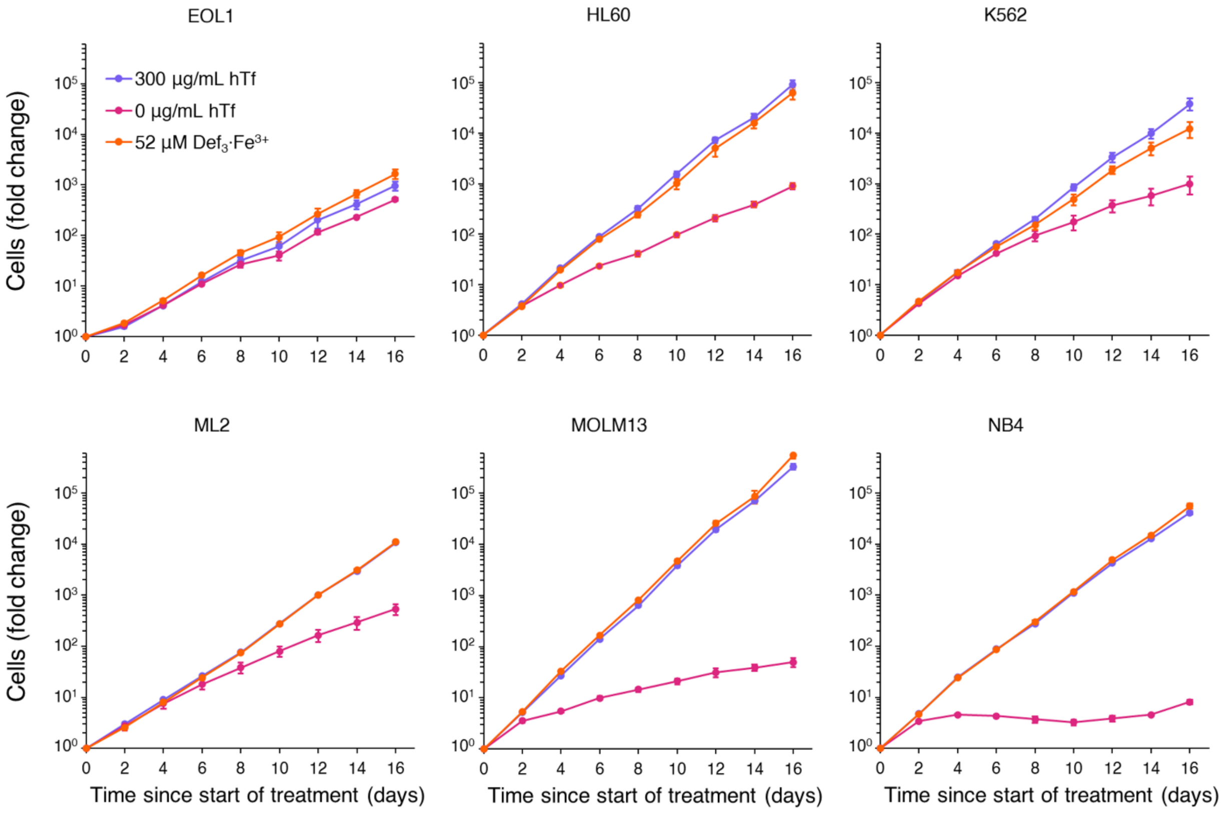

Supplementary Figure S8. Deferiprone sustains expansion of selected myeloid cell lines. Six myeloid cell lines were cultured in serum-free expansion medium supplemented with 300 µg/mL hTf or 52 µmol/L $Def_3{\cdot}Fe^{3+}$ for 16 days. Cells were cultured under static conditions at a concentration of 0.3×10^6 cells/mL, with changing of medium every 2 days. Final cell number fold change relative to day 0 is depicted. Data is displayed as mean ± SD (error bar; n=3).

Supplementary Table S1a. Analysis of the effect of deferiprone supplementation from day 0 of the culture protocol on erythroblast cell numbers.

**Condition**		**Cell number fold change (FC) at day 15 (relative to day 6) (n=3)**		**log_{10}(FC) (n=3)**		
**hTf (µg/mL)**	**$Def_3{\cdot}Fe^{3+}$ (µmol/L)**	**Mean**	**(Range)**	**Mean**	**(Range)**	***p*-value***
300	0	296.6	(227.4 – 357.9)	2.46	(2.36 – 2.55)	
0	0	17.5	(10.4 – 24.9)	1.22	(1.02 – 1.40)	0.0018
30	52	153.5	(106.4 – 233.0)	2.16	(2.03 – 2.37)	0.0399
30	0	36.4	(15.9 – 65.8)	1.49	(1.20 – 1.82)	0.0162
0	52	152.4	(117.3 – 216.1)	2.17	(2.07 – 2.33)	0.0367

Data corresponding to Fig. 4b.

* *p*-values were calculated using the log_{10}(fold change) data and an unpaired 2-tail Student t-test, with the condition "300 µg/mL hTf + no $Def_3{\cdot}Fe^{3+}$" (first row) as reference.

Supplementary Table S1b. Analysis of the effect of deferiprone supplementation from day 7 of the culture protocol on erythroblast cell numbers.

Condition		Cell number fold change (FC) at day 15 (relative to day 7) (n=3)		$\log_{10}$(FC) (n=3)		
**hTf (µg/mL)**	**$Def_3{\cdot}Fe^{3+}$ (µmol/L)**	**Mean**	**(Range)**	**Mean**	**(Range)**	***p*-value***
300	0	96.5	(60.1 – 130.7)	1.96	(1.78 – 2.12)	
0	0	4.5	(2.6 – 5.7)	0.63	(0.41 – 0.76)	0.0008
0	52	41.7	(24.1 – 54.6)	1.59	(1.38 – 1.74)	0.0657
30	0	8.2	(5.8 – 11.5)	0.89	(0.76 – 1.06)	0.0013
30	52	54.0	(32.4 – 65.2)	1.71	(1.51 – 1.81)	0.1479
30	26	66.7	(42.7 – 82.2)	1.81	(1.63 – 1.91)	0.3047
30	13	68.1	(44.6 – 81.3)	1.82	(1.65 – 1.91)	0.3262
30	6.5	56.1	(39.1 – 66.0)	1.74	(1.59 – 1.82)	0.1393
30	3.2	31.0	(21.4 – 36.2)	1.48	(1.33 – 1.56)	0.0174
30	1.6	23.5	(15.3 – 28.1)	1.36	(1.18 – 1.45)	0.0097
30	0.8	17.3	(14.8 – 21.6)	1.23	(1.17 – 1.33)	0.0028

Data corresponding to Fig. 4c and d.

* *p*-values were calculated using the $\log_{10}$(fold change) data and an unpaired 2-tail Student t-test, with the condition "300 µg/mL hTf + no $Def_3{\cdot}Fe^{3+}$" (first row) as reference.

Supplementary Table S1c. Analysis of the effect of deferiprone supplementation from day 7 of the culture protocol on erythroblast viability.

Condition		% of DRAQ7^{-} cells at day 15 (n=3)		
**hTf (µg/mL)**	**$Def_3 \cdot Fe^{3+}$ (µmol/L)**	**Mean**	**(Range)**	***p*-value***
300	0	82.7	(75.4 – 87.5)	
0	0	40.9	(29.1 – 51.2)	0.0049
0	52	82.6	(79.4 – 84.6)	0.9938
30	0	54.5	(40.8 – 64.2)	0.0240
30	52	81.3	(80.0 – 82.9)	0.7370
30	26	81.0	(78.6 – 82.2)	0.6842
30	13	81.9	(79.1 – 84.5)	0.8640
30	6.5	81.6	(78.9 – 83.4)	0.8065
30	3.2	77.7	(72.1 – 81.4)	0.3508
30	1.6	71.5	(66.3 – 76.5)	0.0769
30	0.8	76.5	(67.2 – 87.8)	0.4306

Data corresponding to Fig. 4e.

* *p*-values were calculated using the log_{10}(fold change) data and an unpaired 2-tail Student t-test, with the condition "300 µg/mL hTf + no $Def_3 \cdot Fe^{3+}$" (first row) as reference.
